# Acetylation of p65^Lys310^ by p300 in macrophages mediates anti-inflammatory property of berberine

**DOI:** 10.1016/j.redox.2023.102704

**Published:** 2023-04-17

**Authors:** Shuchen Zhang, Pingyuan Xu, Ziwei Zhu, Lingyan Zhou, Jiao Li, Ruonan Zhou, Yue Kan, Yaru Li, Xizhong Yu, Juan Zhao, Yu Jin, Jing Yan, Penghua Fang, Wenbin Shang

**Affiliations:** a Department of Endocrinology, Jiangsu Province Hospital of Chinese Medicine, The Affiliated Hospital of Nanjing University of Chinese Medicine, Nanjing, 210029, China; b Key Laboratory for Metabolic Diseases in Chinese Medicine, Nanjing University of Chinese Medicine, Nanjing, 210023, China; c School of Chinese Medicine, School of Integrated Chinese and Western Medicine, Nanjing University of Chinese Medicine, Nanjing, Jiangsu, China

**Keywords:** Berberine, p65, p300, Inflammatory response, Macrophages

## Abstract

Nuclear factor (NF)-κB plays a pivotal role in the regulation of inflammatory response in macrophages. Berberine (BBR), which is an active constituent isolated from *Coptis rhizome*, possesses a prominent anti-inflammatory activity. Here we show that BBR changes the global acetylation landscape in LPS-induced protein acetylation of macrophages and reduces the acetylation of NF-κB subunit p65 at site Lys310(p65^Lys310^), leading to the inhibition of NF-κB translocation and transcriptional activity to suppress the expressions of inflammatory factors. BBR resists the inflammatory response in acute LPS-stimulated mice through downregulation of p65^Lys310^ acetylation in peritoneal macrophages. In obese mice, BBR alleviates the metabolic disorder and inflammation with the reduced acetylation of p65^Lys310^ in white adipose tissue. Furthermore, we demonstrate that BBR acts as a regulator of p65^Lys310^ by inhibiting the expression of p300 in macrophages. Our findings elucidate a new molecular mechanism for the anti-inflammatory effect of BBR via the p300/p65^Lys310^ axis.

## 1 Introduction

Berberine (BBR) is a quaternary isoquinoline-based alkaloid, an active constituent isolated from *Coptis rhizome,* which has been used in traditional Chinese medicine for a long time [1]. BBR possesses multispectral therapeutic applications, such as for cardiovascular and metabolic diseases, neurodegenerative disorders, gut infections, and cancer [[2], [3], [4]]. The anti-inflammatory activity is the most prominent characteristic of BBR, which have been widely observed both *in vitro* and *in vivo* [[5], [6], [7], [8]]. The inhibition of inflammation by BBR in multiple types of immune cells is an important common mechanism in the treatment of acute inflammatory diseases as well as a cluster of chronic diseases [[9], [10], [11]]. BBR suppresses the inflammatory response by regulating inflammatory signaling pathways, mainly including nuclear factor κB (NF-κB) pathway, to reduce the production of pro-inflammatory cytokines, such as TNF-α, IL-6, IL-1β and IFN-γ [6]. However, detailed mechanisms for the anti-inflammatory activities of BBR still need further investigation.

In recent years, it has been reported that BBR's multiple pharmacological activities involve a variety of epigenetic changes, such as methylation of DNA, methylation and acetylation of histone, and acetylation of non-histone proteins [12,13]. Epigenetic modifications in immune cells regulate the transcription of proinflammatory cytokines and play important roles in the immunomodulatory effects [14,15]. Lysine acetylation on both histone and non-histone proteins induced by histone acetyltransferases (HATs) and histone deacetylases (HDACs) influences a myriad of cellular and physiological processes [16,17]. Macrophages, which exist in all tissues, play important roles in the regulation of the inflammatory process, and the presence of inflammatory macrophages contributes to the pathogenesis of infections and chronic inflammation disorders, such as atherosclerosis, obesity, diabetes, cancer, and autoimmune diseases [18]. Our previous studies have found that BBR suppresses the inflammatory response in adipose tissue and RAW264.7 macrophages. BBR inhibits the expression of proinflammatory factor secretion and the activation of macrophages [[19], [20], [21]]. The change in lysine acetylation regulates the activity, polarization, and inflammatory responses in macrophages [22]. Some studies have shown that BBR regulates protein acetylation in macrophages, hepatocytes, and cancer cells [13,23,24]. These studies suggest that the modification of protein acetylation in macrophages may mediate the BBR-induced anti-inflammatory activity.

In this study, we first compared the changes of lysine acetylation in the LPS-stimulated macrophages after BBR treatment based on their whole-cell proteomes and lysine acetylomes. A significant decrease in acetylation at Lys310 of p65 (p65^Lys310^), one subunit of NF-κB, induced by BBR was identified. Then, we validated that BBR deacetylated p65^Lys310^ to inhibit the inflammatory responses in the macrophages *in vitro* as well as in acute and chronic inflammatory states. Intriguingly, the BBR-induced deacetylation of p65^Lys310^ was accomplished by lowering the high expression of p300, one of the acetylases. Our research sheds new light on the molecular basis of BBR's anti-inflammatory effect.

## 2 Materials and methods

### 2.1 Reagents and materials

Antibodies, chemicals, recombinant proteins and critical commercial assays are listed in Supplementary Table 1.

### 2.2 Animals and group

#### 2.2.1 Experimental design of acute inflammation in mice

8-week-old male C57BL/6 mice were housed in the animal center with ad libitum access to standard food and sterile water under laboratory conditions of temperature 22 ± 2 °C, 12-h light-dark cycles, and relative humidity 50 ± 15%. Then the mice were randomly divided into 3 groups: the normal diet control group (NC, n = 5), the LPS model group (LPS, n = 5) and the LPS model group with BBR (LPS + BBR, n = 5). An intraperitoneal (i.p.) injection of LPS (dissolved in saline) at a dose of 10 mg/kg of body weight was started at the sixth hour before the animal was sacrificed. The mice in the BBR-treated group were administered by gavage at a dose of 40 mg/kg at 6:00 a.m. every day for a duration of one week. Besides, the mice in the control group were treated with a vehicle (dissolved in 0.05% CMC-Na). During the treatment period, the body weight of mice was recorded every day.

To investigate the inhibitive actions of BBR on ac-p65^Lys310^ activity via downregulation of p300, mice were randomly divided into four groups: the LPS model group (LPS, n = 5), the C646 group (C646, n = 5), the C646 group with LPS (C646+LPS, n = 5) and the C646 group with LPS and BBR (C646+LPS + BBR, n = 5). An intraperitoneal (i.p.) injection of LPS (dissolved in saline) at a dose of 10 mg/kg of body weight was started at the sixth hour before the animal was sacrificed. The mice treated with C646 (dissolved in 0.05% CMC-Na) were injected intraperitoneally at a dose of 30 nmol/g of body weight at 6:00 a.m. every day for a duration of two weeks. Within one week before the end of the experiment, the mice treated with BBR (dissolved in 0.05% CMC-Na) were administered by gavage at a dose of 40 mg/kg of body weight at 6:00 a.m. every day for a duration of one week. Besides, mice in the control group were treated with a vehicle (dissolved in 0.05% CMC-Na) or saline. During the treatment period, the body weight of mice was recorded once a week.

#### 2.2.2 Experimental design of chronic inflammation in mice

6-week-old male C57BL/6 mice were fed adaptively for 2 weeks. Sixteen mice were fed a high fat diet (20 kcal% carbohydrates, 20 kcal% protein and 60 kcal% fat; D12492, OpenSource Diets™) for 12 weeks. During 12 weeks, the body weight and food intake of mice were recorded weekly. Then the mice were randomly divided into two groups: the high-fat diet model group (HFD, n = 8) and the high-fat diet model group with BBR (HFD + BBR, n = 8). Besides, a normal diet (60 kcal% carbohydrates, 21 kcal% protein and 19 kcal% fat; D12450J, OpenSource Diets™) control group (NC, n = 8) was set up. The mice in the BBR-treated group were administered by gavage at a dose of 40 mg/kg of body weight at 6:00 a.m. every day for four weeks duration, while mice in both control groups were treated with a vehicle. During the four-week treatment period, the body weight of mice was recorded once a week. The food intake of all mice was measured once a week. After 3 weeks of treatment, all mice were fasted for 12 h and used for glucose tolerance tests and insulin tolerance tests as described below. At the end of the experiment, fasting blood glucose content in the tail vein blood was measured with a glucometer (Contour TS Blood Glucose Meter, Ascensia Diabetes Care).

All animals received human care and all study protocols were approved by the Animal Studies Committee of Nanjing University of Chinese Medicine.

### 2.3 Glucose tolerance and insulin tolerance tests

While mice were maintained on fasting conditions for 12 h, a glucose solution (1.5 g/kg of mice) was given by i.p. and blood glucose levels were monitored by pricking the tail vein at 0, 15, 30, 60, 90, and 120 min through the glucometer. After 3 days of recovery feeding, we performed insulin tolerance tests. After fasting for 6 h, all animals were intraperitoneally injected with insulin (1 U/kg). The glucose levels in the tail vein blood were quantified at 0, 15, 30, and 60 min after the insulin challenge using the glucometer. The areas under the curve (AUC) were calculated according to the formula [(BG 0 min + BG 30 min) × 1/4 + (BG 30 min + BG 60 min) × 1/4 + (BG 60 min + BG 120 min) × 1/2].

### 2.4 Collection of blood samples and tissues

After fasting for 12 h, all animals were sacrificed on the second day. Then 1 mL of blood, liver, and adipose tissue were quickly collected. In brief, the blood samples were collected in prechilled EDTA tubes containing 100 μL of protease and phosphatase inhibitors (1 μg/mL) and immediately centrifuged for 10 min at 3500 rpm at 4 °C within 30 min of collection. Serum was separated into vials and stored at −80 °C until measurement. The peritoneal macrophages were extracted and stored at −80 °C for further analysis. The liver tissues were rinsed and weighed. The epididymal adipose tissues were harvested, rinsed, and weighed rapidly, and a portion of the adipose tissue was fixed in 4% paraformaldehyde. The rest of the adipose tissue was frozen and stored at −80 °C for further analysis.

### 2.5 Cell culture

#### 2.5.1 RAW264.7 cell line

A murine RAW264.7 macrophage cell line was bought from the Cell Bank of the Chinese Academy of Sciences (Shanghai, China) and cultured in high-glucose DMEM medium supplemented with 10% FBS, 100 U/ml penicillin, and 100 μg/ml streptomycin. RAW264.7 macrophages were acclimated for 12 h and then stimulated with LPS (100 ng/ml) for 24 h. Later, the medium was replaced with DMEM containing 5 μM berberine dissolved in DMSO for 24 h. DMSO was present in the control culture at a concentration of 0.1%. Finally, the culture plates were washed with cold PBS, and the cells were collected with a cell scraper into a centrifuge tube and stored at −80 °C for the following experiments. The experiments were repeated three times.

#### 2.5.2 Primary bone marrow derived macrophage (BMDM)

BMDM were isolated from the thighbone of an 8-week male mouse and cultured in DMEM-conditioned medium with purified recombinant mouse M-CSF for 7 days, changing the medium on the second, fourth, and sixth days of culture, and gradually decreasing the concentration of M-CSF from 20 ng/ml to 5 ng/ml (Supplemental Fig. 1). On the 7th day, when observed under the microscope, the monocytes differentiated into mature macrophages, which could be used for subsequent tests. BMDM macrophages were stimulated with LPS (100 ng/ml) for 24 h. Later, the medium was replaced with DMEM containing 5 μM berberine dissolved in DMSO for 24 h. DMSO was present in the control culture at a concentration of 0.1%. Finally, the culture plates were washed with cold PBS, and the cells were collected with a cell scraper into a centrifuge tube and stored at −80 °C for the following experiments. The experiments were repeated three times.

#### 2.5.3 Primary peritoneal macrophage (PM)

After the acute inflammation animal model was established, to get peritoneal macrophages, the mice were dislocated and killed. These mice were placed on a cold anatomical plate, and the fur was cut along the white line of the abdomen to expose the peritoneum. Inject 5 ml of cold PBS into the peritoneum with a syringe, and gently massage the abdominal area for at least 5 min. Then, carefully cut the peritoneum and suck out all the fluid in the abdominal cavity. Then we collected the fluid into the centrifuge tube, split the red, centrifuged, and sieved it to finally get peritoneal macrophages, which we stored at −80 °C for the following experiments.

### 2.6 Cell viability assay

The CCK8 assay was used to evaluate the effect of berberine on cell viability. Briefly, the RAW264.7 and BMDM cells were cultured in a 96-well plate at a density of 5 × 10^4^ cells per well. After 24 h, cells were treated with different concentrations of berberine (1, 2.5, 5, 10 and 20 μM) for 24 h. After removing the supernatant, 100 μl DMEM with 10 μl CCK8 reagent were added into each well and incubated for 1 h. Then, the absorption values were measured at 450 nm on a microplate reader (EnSpire, USA). The optical density in the control group cells (no berberine) was considered 100% viability.

### 2.7 Lentivirus transfection

Cells were plated in a T25 cell culture flask or six-well plate (1.0 × 10^5^) and cultured overnight before transfection. The cells were transfected with LV-p65, LV-p65 (K310Q), LV-p65 (K310R), or LV-Vector (MOI = 100) (Supplementary Fig. 2) according to the manufacturer's protocols (BrainVTA, Wuhan, China). After 24 h, the cells were washed extensively with phosphate buffer saline (PBS) to remove lentiviral genomic RNA and fresh medium was added. The cells were maintained in culture for another 48 h. Due to the puromycin resistance gene on lentivirus, the fresh medium containing 5 μg/ml puromycin was replaced, and the cells that were not successfully transfected were eluted. Expand the culture of the remaining cells in the flask to form a transfected cell line, which can be used in the follow-up experiment.

### 2.8 Cell transfection with siRNA

The small interfering RNA (siRNA) duplexes for p300 were commercially synthesized by Genepharma (Shanghai, China). The sequences were 5′-GAUGCAGCCUCGAAACAUATT-3′, 5′-GCCUUGGUCUCCAAAUUCATT-3′ and 5′-CUAGCCGAGAAGAUCUAUATT-3′. On the day before transfection, RAW264.7 cells were seeded into six-well plates and grown in 2 ml of DMEM supplemented with 10% FBS. Then, p300 siRNA or scrambled siRNA was transfected into RAW264.7 cells with Lipofectamine RNAiMAX reagent according to the manufacturer's instructions. After 72 h of growth, RAW264.7 cells were used for the further interventions. Meanwhile, the knockdown in p300 expression was evaluated by Western blot analysis.

### 2.9 Quantitative real-time reverse transcriptase polymerase chain reaction (qRT-PCR)

The mRNA expression of MCP-1, IL-1β, and TNF-α were measured by qPCR, and β-actin was used as an internal control for mRNAs. Total RNA was isolated from RAW264.7 cells with Trizol reagent according to the manufacturer's instructions and reverse transcribed into cDNA using the PrimerScript RT Reagent Kit. PCR amplification was performed with a SYBR Green PCR kit using the Applied Biosystems 7500 Real-Time PCR System in triplicates three times independently. The following primer sequences were used (Supplementary Table 2). The parameters of PCR were: 5 min at 95 °C for one cycle, 15 s 95 °C, 1 min 60 °C for 40 cycles, and 5 min 72 °C for one cycle. Reaction specificity was controlled by post-amplification melting curve analyses and gel electrophoresis of products. The expression fold-changes were analyzed by the 2^−^^ΔΔCt^ relative quantitative method.

### 2.10 Subcellular protein fractionation

According to the instructions of the Subcellular Protein Fractionation Kit, we stepwise separate and prepare cytoplasmic, membrane, nuclear soluble, chromatin-bound, and cytoskeletal protein extracts from mammalian cultured cells. The first reagent added to a cell pellet causes selective cell membrane permeabilization, releasing soluble cytoplasmic contents. The second reagent dissolves serum membranes, mitochondrial membranes, and ER/Golgi membranes but not nuclear membranes. After recovering the intact nuclei by centrifugation, a third reagent yields the soluble nuclear extract. A second nuclear extraction with micrococcal nuclease is performed to release chromatin-bound nuclear proteins. The recovered insoluble pellet is then extracted with the final reagent to isolate cytoskeletal proteins.

### 2.11 Co-immunoprecipitation (Co-IP) and immunoblotting

Whole cells were harvested and suspended in an ice-cold lysis buffer. Lysates were sonicated on ice and incubated for 30 min before being centrifuged at 12,000 rpm for 30 min at 4 °C. Protein concentration was determined using the BCA protein assay kit. For Co-IP analysis, lysates were pre-cleared with protein A/G agarose beads for 1 h. Pre-cleared samples were then incubated with indicated antibodies overnight and protein A/G agarose beads for 1 h at 4 °C, or with antibody/glutathione-conjugated agarose for 4 h at 4 °C. The agarose beads were washed extensively, and samples were eluted by boiling at 95 °C for 10 min. Precipitated proteins were analyzed by SDS gel electrophoresis and immunoblotting.

For immunoblotting analysis, equal amounts of protein were separated by 12% SDS-PAGE and transferred to PVDF membranes (Millipore, Bedford, MA, USA). Membranes were incubated with blocking buffer (5% w/v BSA in TBS containing 0.1% Tween-20) for 2 h at room temperature and with a primary antibody overnight at 4 °C. After washing with TBS containing 0.1% Tween-20, the membranes were incubated for 2 h at room temperature with HRP-linked secondary antibodies. The membranes were then detected by the ECL plus western blotting detection.

### 2.12 Enzyme-linked immunosorbent assay (ELISA)

A double-antibody sandwich ELISA was used. The specific anti-mouse antibody was pre-coated on the high-affinity enzyme plate. The standard was added to the air on the enzyme plate. After incubation, the proteins in the samples bind to the solid-phase antibodies and detection antibodies. After washing and removing unbound substances, add streptavidin-HRP. After washing, add the chromogenic substrate TMB to avoid light and develop color. The depth of the color reaction is proportional to the protein concentration in the sample. The terminating solution was added to terminate the reaction, and the absorbance was determined at 450 nm (reference wavelength: 570–630 nm).

### 2.13 Immunofluorescence (IF)

The cells cultured in 96-well plates were washed once with aseptically preheated PBS, then 4% PFA precooled by 50 μL was added to each well and fixed at room temperature for 30 min, then washed with PBS 3 times for 5 min each time. 0.1% Triton X-100 of 200 μL was added to each well and incubated at room temperature for 10 min. Then rinse with PBS three times for 5 min each. Add 5% BSA in the amount of 200 μL to each well, seal at room temperature for 90 min, discard the sealing solution, add a diluted antibody of 1:200, and incubate at 4 °C overnight. PBS was washed three times for 5 min each time, and a diluted fluorescent secondary antibody of 1:500 was added to avoid light and incubated at room temperature for 2 h. PBS was washed 3 times for 5 min each time, DAPI was added and incubated at room temperature for 10 min, DAPI was discarded, and PBS was washed 3 times for 5 min each time. Finally, retain 200 μL of PBS in each well and analyze it by high content screening (HSC) on Operetta (PerkinElmer).

### 2.14 NF-κB transcription activity assay

According to the manufacturer's instructions, an NF-kB p65 Transcription Factor Assay Kit was performed to detect NF-κB p65 DNA-binding activity. The absorbance at 450 nm was determined using a microplate reader.

### 2.15 Proteomics and acetyl-proteomics

All proteomics and acetyl-proteomics were performed by PTM-Biolabs (Hangzhou, China). Cell samples were lysed, and the protein was extracted in lysis buffer (8 M urea, 1% protease inhibitor cocktail, 3 μM TSA, and 50 mM NAM for acetylation). After determining the protein concentration, the protein sample was added to trypsin at a 1:50 and 1:100 trypsin-to-protein mass ratio for digestion. Then, the mass of peptides was labeled by TMT and fractionated by HPLC. Tryptic peptides were incubated with pre-washed antibody beads for pan antibody-based PTM enrichment and then carried out by high-resolution liquid chromatography–mass spectrometry (LC–MS/MS). The resulting LC–MS/MS data were processed using the Maxquant search engine (v. 1.5.2.8). Tandem mass spectra were searched against the human Uniprot database concatenated with the reverse decoy database. Protein quantitative normalization is used to eliminate the effect of protein expression on modification abundance, which can be used in subsequent bioinformatics analysis. The screening of differential modification sites followed the following criteria: 1.3 times the change threshold, *t*-test *p* < 0.05.

### 2.16 Statistical analysis

Statistical analyses were performed using GraphPad Prism 9.0. All values were expressed as mean ± standard errors of the means (SEMs). All the data were from at least three independent experiments. Differences among multiple groups were determined by one-way analysis of variance (ANOVA) followed by Tukey's post-hoc test. Values were considered significantly different at *p* < 0.05.

## 3 Results

### 3.1 BBR ameliorates LPS-induced inflammation and changes total acetylated-lysine level in macrophages

**Fig. 1:**
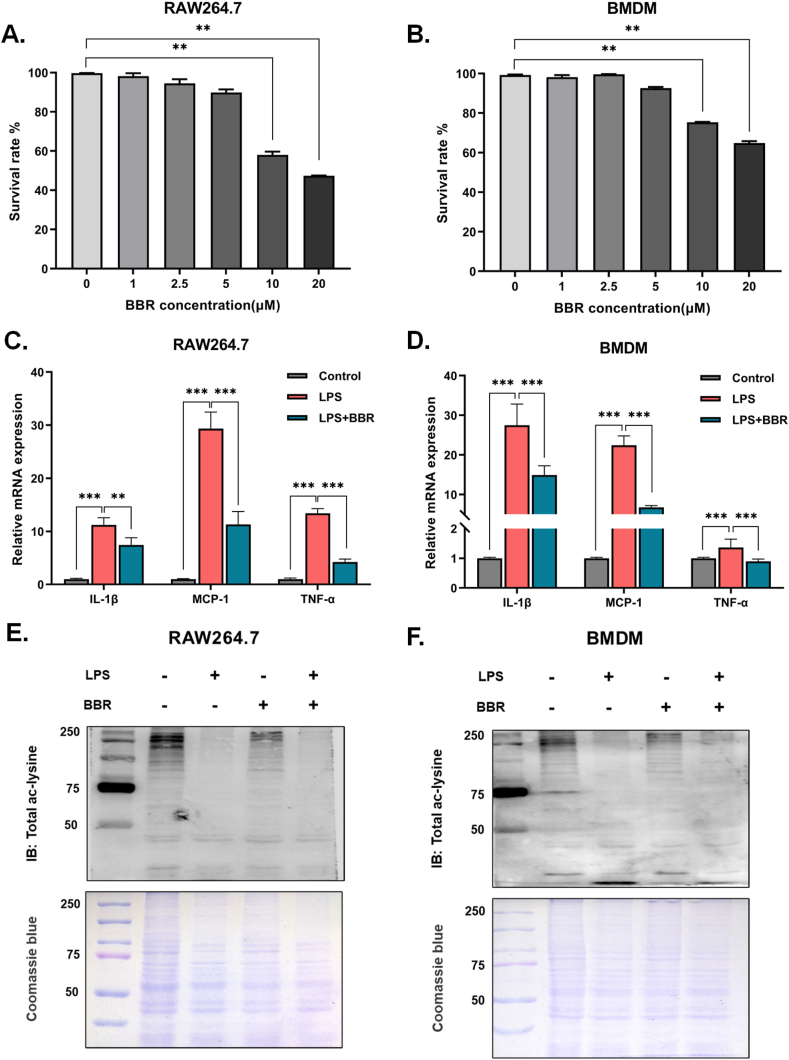
BBR reduced the inflammation stimulated by LPS and regulated total protein acetylation in macrophages **A-B.** The RAW264.7 and BMDM cells were treated with different concentrations of BBR for 24 h. The CCK8 assay kit was then used to detect the cell survival rate, and the relative cell viability was calculated by comparing it to the untreated control group. **C-D.** Effect of BBR (5 μM) on inflammatory gene expression (IL-1β, MCP-1 and TNF-α) in RAW264.7 and BMDM cells after pretreatment with or without LPS (100 ng/ml) for 24 h (n = 3). **E-F:** Effect of BBR (5 μM) on pan-acetylated protein in RAW264.7 and BMDM cells after pretreatment with or without LPS (100 ng/ml) for 24 h. Coomassie brilliant blue staining was used as a loading control. The data collected are expressed as the mean ± SEM for the gene expression. *p < 0.05 & **p < 0.01 & ***p < 0.001. (For interpretation of the references to color in this figure legend, the reader is referred to the Web version of this article.)

To investigate the anti-inflammatory effect of BBR, we examined the expression levels of inflammatory genes in LPS-stimulated RAW264.7 cells and bone marrow-derived macrophages (BMDM). First, we determined the effect of BBR on viability in RAW264.7 and BMDM (Fig. 1A–B). BBR showed cytotoxicity in both RAW264.7 and BMDM cells when the concentration was higher than 5 μM in CCK8 assays, suggesting that the optimal dose of BBR is 5 μM *in vitro*. Subsequently, we examined the anti-inflammatory effect of BBR *in vitro*. The results showed that the mRNA levels of inflammatory factors (IL-1β, MCP-1 and TNF-α) were markedly decreased after treatment with 5 μM BBR for 24 h in both LPS-stimulated RAW264.7 and BMDM cells (Fig. 1C–D). These findings suggest that BBR exerts an anti-inflammatory effect in macrophages through inhibition of proinflammatory cytokines.

Previous reports have indicated that the expression of inflammatory factors in macrophages is closely related to protein acetylation, which is a common and widespread post-translational modification of proteins [25]. To investigate the inhibitive effect of BBR on inflammatory factors, we examined the effects of BBR on pan-acetylated protein levels in both LPS-stimulated RAW264.7 macrophages and BMDM. Interestingly, we found that the acetylation level of total proteins in LPS-induced macrophages was slightly increased after treatment with BBR (Fig. 1E–F), although the level of total pan-acetylated proteins distinctly decreased in both LPS-stimulated RAW264.7 and BMDM macrophages. These results suggest that BBR could change the acetylation level of intracellular proteins in LPS-induced macrophages, which may be closely related to its anti-inflammatory effect.

### 3.2 BBR decreases acetylation of p65^Lys310^ to suppress NF-κB activity

**Fig. 2:**
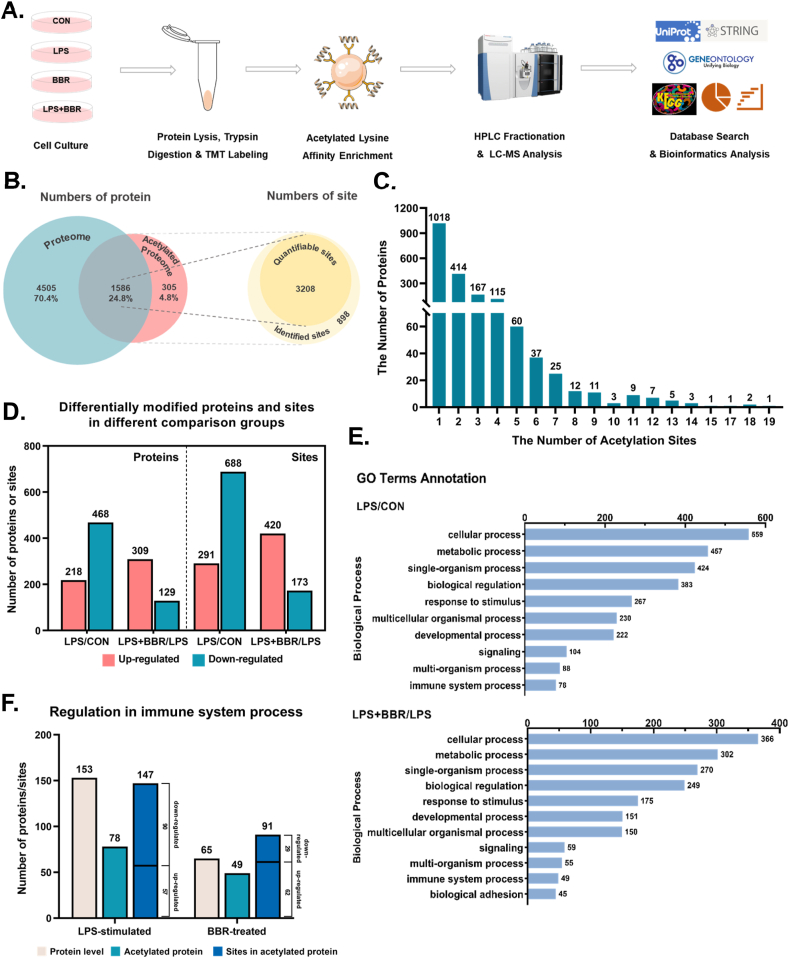
Global landscape and functional annotation of BBR-regulated acetylation in macrophages. **A.** Schematic representation of the experimental workflow for TMT quantification of Kac in RAW264.7 cells. **B.** Comparison of overlapped acetylated proteins between the total proteome and the total acetylated proteome. Values are the number of proteins (blue and red) or sites (yellow), respectively. **C.** The distribution of the number of identified Kac sites per protein. **D.** Statistics and comparison of proteome and acetylated proteome between CON, LPS and LPS + BBR group specifically. **E.** The GO terms annotate biological process in acetyl-proteome. Here is a list of the top 10 or 11 pathways. **F.** The changes of proteins, acetylated proteins and acetylated sites among immune process after GO terms annotation. (For interpretation of the references to color in this figure legend, the reader is referred to the Web version of this article.)

To obtain a global view of the BBR-regulated acetylated proteome, especially Kac of nonhistone substrates, we used a series of approaches involving TMT labeling, high-performance liquid chromatography (HPLC) fractionation, immunoaffinity enrichment, and high-resolution LC-MS/MS to investigate Kac substrates in RAW264.7 cells with or without LPS or BBR (Fig. 2A and Supplementary Fig. 3). In brief, among a total of 4106 Kac sites across 1891 proteins in the total proteome, 3208 Kac sites from 1586 proteins were identified in the acetylated proteome (Fig. 2B). The proteome and acetylated proteome were counted separately, and the trend in quantity of different sites compared in the two groups was similar (Supplemental Table 3). Among all acetylated proteins, approximately 53.8% contained only one acetylated lysine site, and the percentages of proteins with two, three, four, or >4 modification sites of lysine were 21.8%, 8.8%, 6.1%, and 9.5%, respectively (Fig. 2C). Furthermore, the results showed that 688 acetylated lysine sites of 468 proteins were downregulated, while 291 acetylated lysine sites of 218 proteins were upregulated in all 3 cases of LPS-stimulated RAW264.7 macrophages compared to those in control groups (Fig. 2D). Compared with LPS-stimulated groups, 420 acetylated lysine sites on 309 proteins were upregulated, while 173 acetylated lysine sites on 129 proteins were downregulated in all 3 cases of BBR-treated RAW264.7 macrophages (Fig. 2D).

Acetylation of non-histone proteins has been shown to have abundant functions [16]. To determine the effect of lysine acetylation on biological processes, gene ontology enrichment analysis was also performed to identify different groups of functionally related acetylated proteins according to cell component, molecular function, and biological process (Supplemental Fig. 4). Furthermore, the criteria of >1.3 or <0.769, *t*-test p-value<0.05 as an up-regulation or down-regulation of the lysine acetylation site, was recognized as a significant difference among expressed lysine acetylation sites. Within the LPS group or BBR group of biological processes, there was enrichment for those involved in cellular processes, metabolic processes, immune system processes, etc. (Fig. 2E). Among the immune system processes, 78 proteins with 147 lysine sites were changed in the LPS group, while LPS-induced changes in the immune system processes were switched to the 91 lysine sites on 49 proteins after treatment with BBR (Fig. 2F). All significant ontology terms for up- and downregulated lysine acetylation in immune processes are detailed in Supplemental Table 4. Of these lysine sites, 62 were upregulated and 29 were downregulated with BBR treatment after LPS stimulation (Fig. 2F). The trend of lysine acetylation level seemed similar to the result of pan-acetylation level by Western blot (Fig. 1E–F). Therefore, these results demonstrated that BBR changed the global acetylation landscape in LPS-induced protein acetylation in macrophages.

**Fig. 3:**
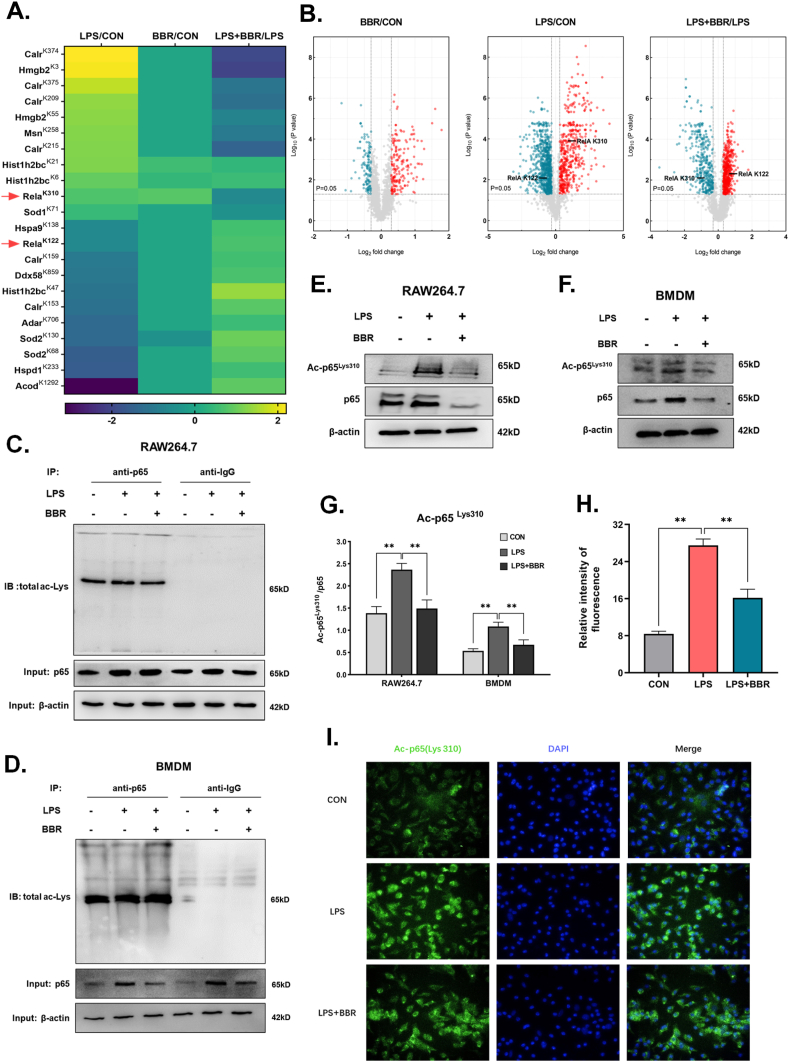
Berberine suppressed acetylation of p65 at site Lys310 in macrophages. **A.** The heatmap of acetyl-sites in inflammatory process through the enrichment of KEGG. **B.** A volcanic diagram of acetylation modification genomics. Each picture is the result of a comparison between the two groups. A dot represents a lysine residue on a protein detected, the abscissa represents the log2 value of the difference multiple of acetylation, and the ordinate represents the p-value. The protein with a difference of more than 1.3 times is selected as the positive result. Blue represents a decrease in acetylation level, red represents an increase, and gray represents no significant difference. **C-D.** The immunoprecipitation analysis of the total acetylation level of p65 in RAW264.7 and BMDM cells. The representative Western blot lines of total acetyl-p65 and p65 proteins in RAW264.7 and BMDM cells. The result of the anti-IgG was indicated as a negative control. β-actin was used as the loading control. **E-F.** The representative Western blot lines of ac-p65^Lys310^ and p65 proteins in RAW264.7 and BMDM cells after treatment with BBR in the presence of LPS. The result of the anti-IgG was indicated as a negative control. β-actin was used as the loading control. **G.** The protein level of ac-p65^Lys310^ in RAW264.7 and BMDM cells after treatment with BBR in the presence of LPS (n = 3). **H.** The intensity of ac-p65^Lys310^ immunofluorescence in BMDM cells after treatment with BBR in the presence of LPS (n = 3). The nuclear dye DAPI was used as the internal reference. **I.** The photomicrographs of ac-p65^Lys310^ immunofluorescence in BMDM cells after treatment with BBR in the presence of LPS. All data shown are the means ± SEM. **p < 0.01. (For interpretation of the references to color in this figure legend, the reader is referred to the Web version of this article.)

There was an overrepresentation of several pathways known to be regulated by BBR [19], including metabolism of glucose and regulation of the pro-inflammatory transcription factor NF-κB. According to the results of the acetylated proteome above mentioned (Fig. 2F), the acetylation of two lysine sites in NF-κB with a significant change was altered in the LPS-induced macrophages after BBR treatment, which are located at Lys310 and Lys122 (Fig. 3A). As ac-p65^Lys310^ is important in the regulation of inflammation, we focused on the dynamic change and function of p65 acetylation on Lys310. According to the volcano plot of the acetylated proteome (Fig. 3B), the degree of ac-p65^Lys310^ increased dramatically in LPS-induced macrophages but decreased significantly in BBR-treated macrophages. To confirm these results, the levels of total ac-p65 and ac-p65^Lys310^ were validated in both BBR-treated RAW264.7 and BMDM macrophages. The results showed that the total acetylation of p65 was not distinctly changed in the LPS-induced group or the BBR-treated group compared with the control group (Fig. 3C–D). However, the levels of ac-p65^Lys310^ and p65 were strongly decreased in BBR-treated RAW264.7 and BMDM macrophages after LPS stimulation compared with the LPS control group, whereas they were distinctly upregulated in LPS-stimulated RAW264.7 and BMDM macrophages compared with the control group (Fig. 3E–G). Consistent with the results above, morphological results also showed that the levels of ac-p65^Lys310^ were mostly increased in the LPS-stimulated BMDM macrophages, while they were dramatically reduced after administration of BBR (Fig. 3H and I). Taken together, these findings suggest that BBR decreases the LPS-induced elevation of acetylation of p65^Lys310^ in macrophages.

**Fig. 4:**
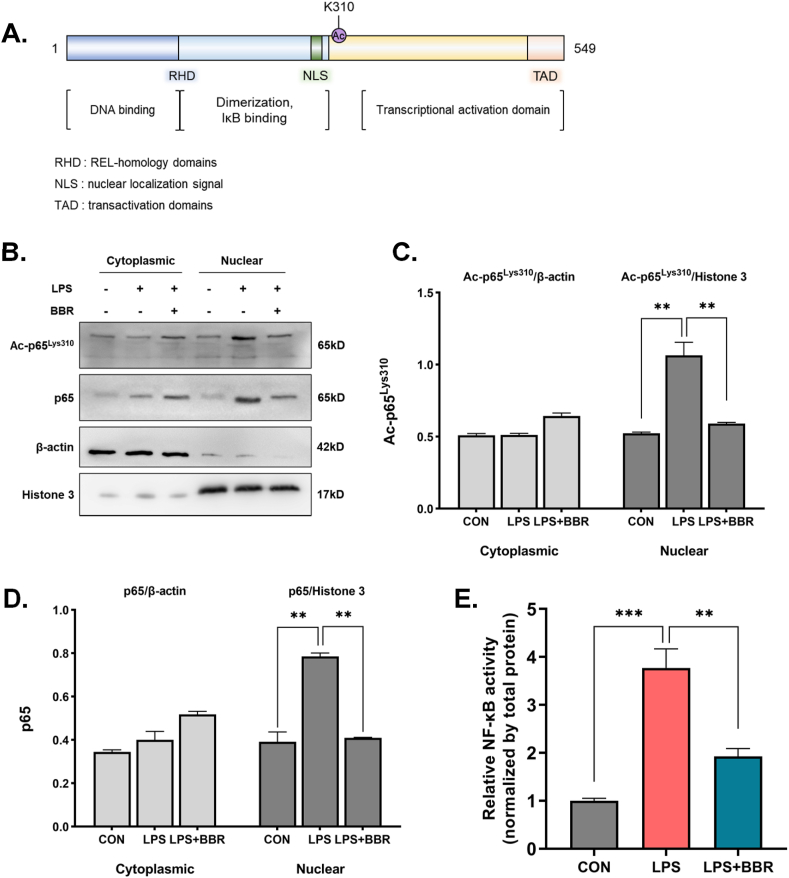
Effect of BBR-inhibited ac-p65^Lys310^ on the activity of NF-κB translocation and transcription **A.** The domain pattern of p65, the linear structure location of the Lys310 site and the physiological function of the domain. **B.** The representative Western blot lines of ac-p65^Lys310^ and p65 proteins in the cytoplasm and nucleus of RAW264.7 cells after treatment with BBR in the presence of LPS. β-actin and histone 3 were used as the loading control. **C-D**. The protein levels of ac-p65^Lys310^ and p65 in the cytoplasm and nucleus of RAW264.7 cells after treatment with BBR in the presence of LPS (n = 3). **E.** The level of NF-κB activity in RAW264.7 cells following BBR treatment in the presence of LPS (n = 3). All data shown are the means ± SEM. **p < 0.01.

Lys310 is located in the transactivation domain of p65 (Fig. 4A) and plays a key role in the regulation of NF-κB activity through promoting the translocation of p65 from the cytoplasm to the nucleus. To further confirm the effect of berberine on NF-κB activity, the levels of nuclear ac-p65^Lys310^ and p65 were detected *in vitro,* respectively. As a result, the levels of ac-p65^Lys310^ and p65 were markedly increased in the extracting of protein from the nucleus of LPS-induced RAW264.7 macrophages compared with the control group (Fig. 4B–D). However, BBR treatment led to a significant reduction of nuclear ac-p65^Lys310^ levels and p65 in the extracting of proteins from the nucleus of LPS-induced RAW264.7 macrophages (Fig. 4B–D). Additionally, we found that the transcriptional activity of NF-κB was decreased after administration of BBR in the LPS-stimulated RAW264.7 macrophages (Fig. 4E). These results suggested that BBR inhibited the translocation of p65 from the cytoplasm to the nucleus and NF-κB activity via downregulation of nuclear ac-p65^Lys310^ in macrophages. Together with these findings, our data suggested that BBR could weaken the activity of NF-κB through reducing levels of ac-p65^Lys310^ to suppress p65 translocation.

### 3.3 BBR loses its anti-inflammation effect after lysine residue mutant of p65^Lys310^

**Fig. 5:**
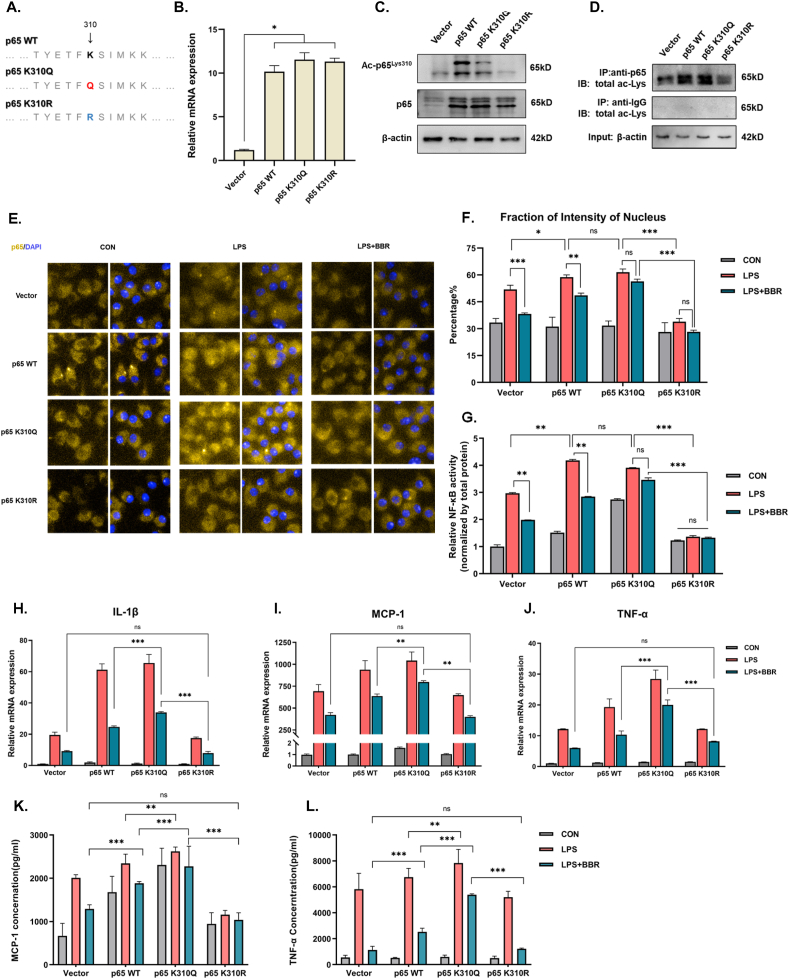
Effect of the mutant of p65 at the site of Lys310 on the anti-inflammation of BBR in macrophages. **A.** The pattern of the p65 mutant at position Lys310 was constructed with lentivirus. Q represents the mutation of lysine to glutamine, which is similar to continuous acetylation, while R represents the mutation of lysine to arginine, which is similar to continuous inhibition of acetylation. **B.** Validation of p65 mRNA levels for transfection efficiency in the p65 wild and mutant groups (n = 3). **C-D.** Validation of ac-p65^Lys310^ and total ac-p65 levels for transfection efficiency in the p65 wild and mutant groups. The representative Western blot lines of ac-p65^Lys310^ and total ac-p65 proteins in the p65 wild and mutant groups. The result of the anti-IgG was indicated as a negative control. β-actin was used as the loading control. **E.** The photomicrographs of ac-p65 immunofluorescence in the nucleus of the p65 wild and mutant RAW264.7 cells after treatment with BBR in the presence of LPS. The nuclear dye DAPI was used as the internal reference. **F.** The intensity of ac-p65 immunofluorescence in the nucleus of the p65 wild and mutant RAW264.7 cells after treatment with BBR in the presence of LPS (n = 3). **G.** The level of NF-κB activity in the p65 wild and mutant RAW264.7 cells following BBR treatment in the presence of LPS (n = 3). **H-J.** The levels of inflammatory gene expression (IL-1β, MCP-1 and TNF-α) in the p65 wild and mutant RAW264.7 cells after BBR treatment in the presence of LPS (n = 3). **K-L.** Inflammatory cytokine levels (MCP-1 and TNF-α) in the supernatant of p65 wild and mutant RAW264.7 cells after BBR treatment in the presence of LPS (n = 3). All data shown are the means ± SEM. *p < 0.05 & **p < 0.01 &***p < 0.001; ns, no significance.

To further confirm whether BBR exerts anti-inflammatory effects via inhibition of p65 acetylation at the site of Lys310, we constructed two cell lines with lysine residue mutations in RAW264.7 cells (Fig. 5A). The lysine mutation to arginine at 310 (K310R) represented continuing inhibition of acetylation, while the lysine mutation to glutamine at 310 (K310Q) represented continuing elevation of acetylation (Supplemental Fig. 2B). The efficiency of lentivirus transfection was verified as shown in Fig. 5B–C. The mRNA and total protein levels of p65 were markedly elevated after lentivirus transfection (Fig. 5B–C). Within the lentivirus transfection groups, the total acetylation of p65 as well as ac-p65^Lys310^ were significantly decreased in the K310R mutant, whereas they were slightly increased in the K310Q mutant compared with the wild type (Fig. 5C–D). These findings suggest that the two cell lines with lysine residue mutations were successfully transfected due to a non-specific antibody for the site-mutated protein. Next, we detected the translocation of p65 from the cytoplasm to the nucleus in the wild-type and p65 lysine residue mutations of RAW264.7 cells following LPS or BBR treatment for 24 h (Fig. 5E). As a result, the total fluorescence intensity of p65 in cells of all transfected groups was significantly higher than that in the vector group (Fig. 5E). Compared with the control group, the total fluorescence intensity of p65 as well as the nuclear fluorescence intensity of p65 were significantly upregulated in all four groups after LPS stimulation, especially in the wild-type and K310Q groups (Fig. 5E–F). Besides, we found that the total fluorescence intensity of p65 as well as the nuclear fluorescence intensity of p65 in the LPS-induced K310Q group were higher than in the LPS-induced vector group, whereas there was no significant change between the LPS-induced K310R group and the LPS-induced vector group (Fig. 5E–F). These results suggest that acetylated Lys310 of p65 strengthened the translocation of p65 from the cytoplasm to the nucleus in the inflammatory process of macrophages. Notably, BBR treatment reversed the LPS-induced elevation of the total p65 fluorescence intensity as well as the nuclear fluorescence intensity of p65 in the wild-type group (Fig. 5E–F). However, the total fluorescence intensity of p65 as well as the nuclear fluorescence intensity of p65 in both the K310Q and K310R groups showed no significant change after BBR treatment (Fig. 5E–F). These results indicate that the BBR-induced reduction of p65 translocation was abolished by a mutant of p65^Lys310^ in both K310Q and K310R cells. Additionally, we further tested the activity of NF-κB with the assay kit (Fig. 5G). The results showed that the activity of NF-κB was markedly reduced in the LPS-induced wild-type group after BBR treatment, while the BBR-induced reduction of activity of NF-κB was also abolished by the mutant of p65 in both K310Q and K310R cells (Fig. 5G). Taken together, these findings suggest that BBR suppresses p65 translocation to reduce the activity of NF-κB through inhibition of acetylation of p65 at site Lys310.

Moreover, we examined the expression and section of inflammatory factors such as IL-1β, MCP-1 and TNF-α in both K310Q and K310R cells. The results showed that the expression and secretion of cytokines (IL-1β, MCP-1 and TNF-α) were markedly increased in the LPS-induced K310Q and wild-type groups, but there was no significant change in the K310R group (Fig. 5H-L), suggesting that acetylated Lys310 in p65 is a necessary site for LPS-induced inflammatory cytokines. More importantly, we found that the expression and section of cytokines (IL-1β, MCP-1 and TNF-α) were markedly decreased in the BBR-treated wild-type group, but there was no significant change in the BBR-treated K310Q or K310R groups (Fig. 5H-L), suggesting that acetylated Lys310 in p65 is a necessary site for BBR-induced reduction of inflammatory factors. Altogether, these findings suggest that BBR exerts an anti-inflammatory effect through reducing the acetylation of p65 at site Lys310 to suppress the activity of NF-κB.

### 3.4 BBR attenuates acute inflammation in macrophages of LPS-induced mice via inactivation of ac-p65^Lys310^

**Fig. 6:**
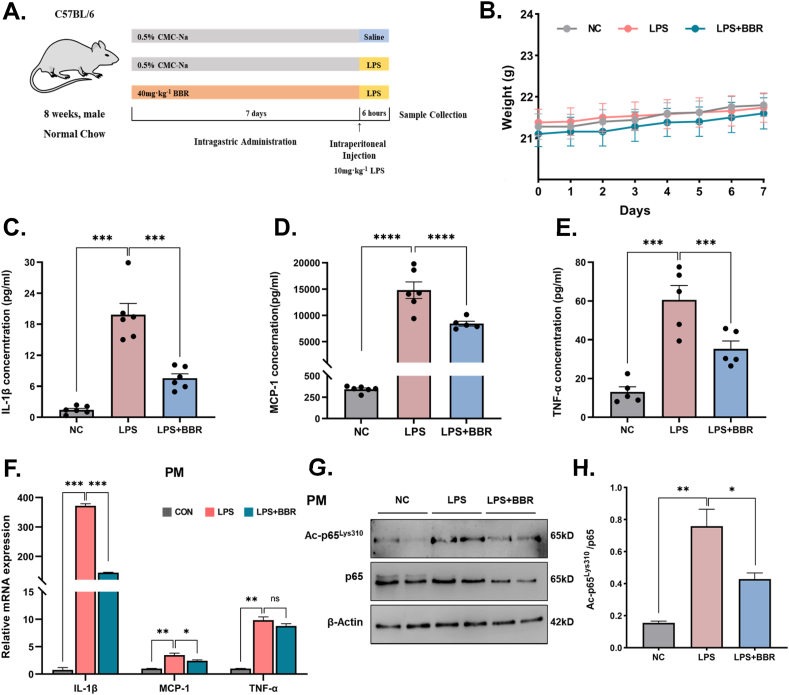
BBR reduced acute inflammation and the level of ac-p65^Lys310^ in macrophages of LPS-induced mice. **A.** Schematic figure illustrating the animal experiment of acute inflammation. **B.** The changes in body weight after BBR treatment in LPS-induced mice (n = 5). **C-E.** The levels of serum IL-1β, MCP-1 and TNF-α after BBR treatment in LPS-induced mice (n = 5). **F.** Inflammatory gene expression levels (IL-1, MCP-1 and TNF-α) in peritoneal macrophages of LPS-induced mice after BBR treatment (n = 3). **G.** The representative Western blot lines of ac-p65^Lys310^ and p65 proteins in peritoneal macrophages of LPS-induced mice after BBR treatment. β-actin was used as the loading control. **H.** The level of ac-p65^Lys310^ in peritoneal macrophages of LPS-induced mice after BBR treatment (n = 3). All data shown are the means ± SEM. *p < 0.05 & ***p < 0.001; ns, no significance.

In order to verify the role of anti-inflammatory BBR through reducing acetylation of p65 at site Lys310 *in vivo*, the mouse model of acute inflammation with short-term LPS treatment was used in the next experiments (Fig. 6A). The short-term treatment with BBR didn't show a remarkable decrease in body weight in mice (Fig. 6B). Furthermore, we analyzed the expression levels of cytokines involved in the proinflammatory process. As a result, short-term LPS stimulation significantly increased serum IL-1β, MCP-1, and TNF-α levels in mice, whereas BBR injection decreased serum IL-1β, MCP-1, and TNF-α levels in mice with LPS-induced inflammation (Fig. 6C–E). In addition, mRNA levels of IL-1β, MCP-1 and TNF-α in peritoneal macrophages were markedly reduced after BBR treatment in mice with LPS-induced inflammation (Fig. 6F), further confirming BBR's anti-inflammatory effect. Moreover, the inhibitory effect of BBR on ac-p65^Lys310^ in macrophages was investigated *in vivo*. As a result, the protein levels of ac-p65^Lys310^ were strongly elevated in peritoneal macrophages of LPS-stimulated mice (Fig. 6G–H). However, BBR treatment led to a significant reduction of ac-p65^Lys310^ levels in peritoneal macrophages of LPS-stimulated mice. Taken together, these findings suggest that BBR could inhibit expression of proinflammatory cytokines via downregulation of ac-p65^Lys310^ in macrophages, which may contribute to the attenuation of acute inflammation in LPS-induced mice.

### 3.5 BBR alleviates chronic inflammation in the adipose tissue of HFD-induced obese mice via inactivation of ac-p65^Lys310^

**Fig. 7:**
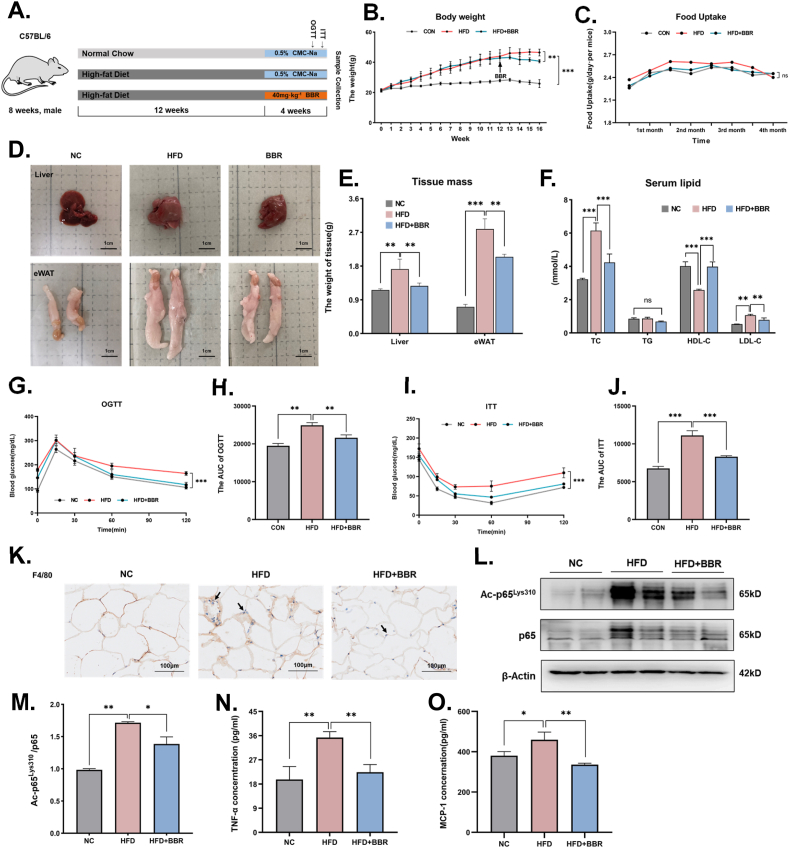
BBR alleviated the metabolic disorder and inflammation in white adipose tissue in high-fat diet-induced obese mice. **A.** Schematic figure illustrating the animal experiment of chronic inflammation. **B.** The changes in body weight after BBR treatment in high-fat diet-induced obese mice (n = 8). **C.** The changes in food intake after BBR treatment in high-fat diet-induced obese mice (n = 8). **D.** Morphological changes of liver and epididymal adipose tissue after BBR treatment in high-fat diet-induced obese mice. **E.** The weight of liver tissue and epididymal adipose tissue after BBR treatment in high-fat diet-induced obese mice (n = 8). **F.** The serum levels of total cholesterol, triglyceride, high-density lipoprotein and low-density lipoprotein after BBR treatment in high-fat diet-induced obese mice (n = 8). **G-H.** A scattered map and histogram show the GTT levels of the high-fat diet-induced obese mice after BBR treatment (n = 5). **I-J.** A scattered map and histogram show the ITT levels of the high-fat diet-induced obese mice after BBR treatment (n = 5). **K.** The photomicrographs of F4/80 expression and macrophage infiltration in white adipose tissue of the high-fat diet-induced obese mice after BBR treatment, **L.** The representative Western blot lines of ac-p65^Lys310^ and p65 proteins in white adipose tissue of the high-fat diet-induced obese mice after BBR treatment. β-actin was used as the loading control. **M.** The protein level of ac-p65^Lys310^ in white adipose tissue of the high-fat diet-induced obese mice after BBR treatment (n = 3). **N–O.** The levels of serum TNF-α and MCP-1 in the high-fat diet-induced obese mice after BBR treatment (n = 5). AUC: area under the curve. All data shown are the means ± SEM. *p < 0.05 & **p < 0.01 & ***p < 0.001; ns, no significance.

NF-κB is important not just in the acute inflammatory signaling pathway but also in chronic inflammation. Obese mice are thought to exhibit a state of continuous low-grade inflammation. In the following study, BBR was given to high-fat-induced obese mice to see if the pharmacological action of BBR on chronic inflammation is also mediated by modifying the acetylation of p65^Lys310^ (Fig. 7A). As expected, BBR treatment significantly reduced body weight in mice, which is consistent with the previous report's findings of decreased adipose tissue and body weight after BBR treatment in high-fat diet obese mice [[26], [27], [28]](Fig. 7B). However, the daily food intake was not significantly different between the LPS-combining BBR-treated mice and those in the LPS group, suggesting that BBR-induced reductions in body weight did not stem from effects on the reduction of food intake (Fig. 7C). These results suggest that BBR can help mice lose weight without reducing their food intake. Besides, the weight of the liver and white adipose tissue was reduced after treatment with BBR, suggesting that BBR minimized the increase in liver and eWAT caused by HFD, mostly by reducing lipid deposition (Fig. 7D–E). In addition, the total cholesterol (TC) and low-density lipoprotein cholesterol (LDL-C) of HFD mice increased in varying degrees, while the high-density lipoprotein cholesterol (HDL-C) decreased slightly (Fig. 7F). However, the change in triglyceride (TG) was not significant, which was consistent with the blood lipid changes of hyperlipidemia. Treatment with BBR in the mice improved all parameters except for the change in TG, demonstrating that BBR has a good hypolipidemic effect. Furthermore, the glucose tolerance and insulin sensitivity of the mice treated with BBR were much better than those of obese control mice (Fig. 7G–J), suggesting that BBR improved aberrant blood glucose levels, glucose tolerance, and insulin sensitivity in obese mice.

Under normal conditions, the extracellular matrix (ECM) of eWAT contains few or no macrophages. Macrophages are recruited to adipose tissue after HFD modeling, and a high number of macrophages can be detected in the ECM that are intimately connected to one another (Fig. 7K). The quantity of macrophages in the ECM was reduced dramatically and showed a dispersed distribution after BBR treatment, demonstrating that BBR can diminish macrophage recruitment in eWAT during chronic inflammation (Fig. 7K). Furthermore, the levels of ac-p65^Lys310^ in eWAT increased considerably following HFD modeling but decreased after BBR treatment (Fig. 7L-M). Moreover, pro-inflammatory factors such as TNF-α and MCP-1 were detected in serum using an ELISA kit (Fig. 7N-O). As a result, TNF-α and MCP-1 secretion were elevated in the serum of obese mice, whereas BBR dramatically reduced TNF-α and MCP-1 secretion. Taken together, these data suggest that BBR reduced macrophage recruitment in eWAT and decreased the expression of proinflammatory cytokines by downregulating ac-p65^Lys310^ in eWAT macrophages, which helped reduce chronic inflammation in HFD-induced animals.

### 3.6 BBR reduces acetylation level of Lys310 via regulating p300 expression

**Fig. 8:**
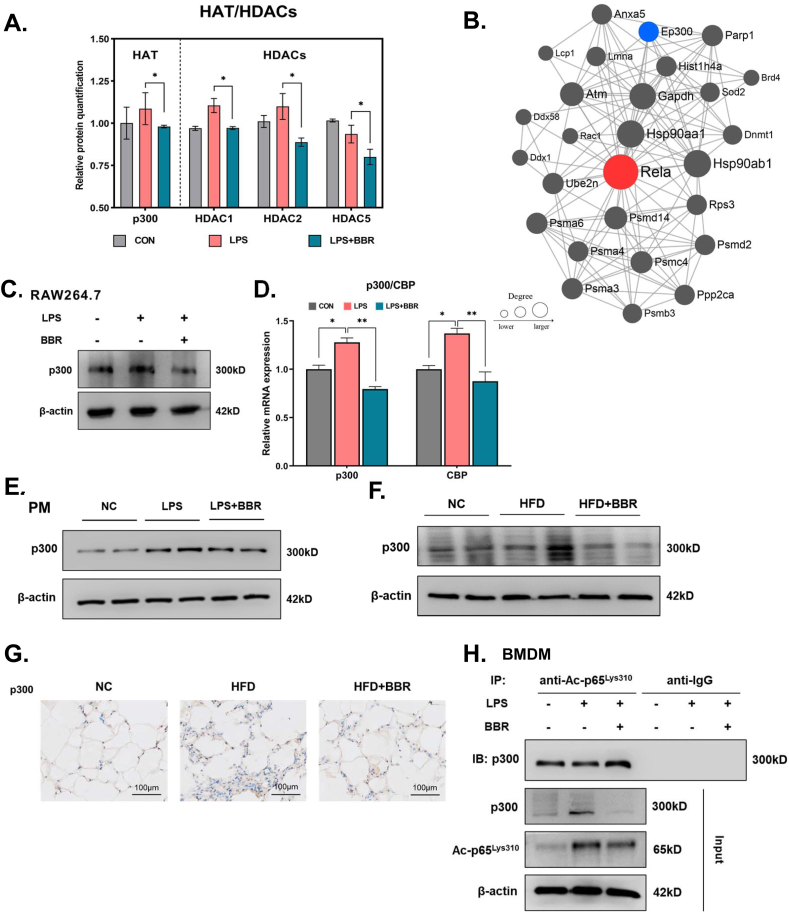
**BBR reduced the level of ac-p65**^**Lys310**^**via regulating p300 expression in macrophages.** **A.** According to screening from proteomics, BBR treatment led to acetylase and deacetylase with significantly different changes in macrophages. **B.** Based on the STRING protein database, the PPI map centered on p65 was drawn according to the modification group data. Red is p65 protein, and blue is p300 protein. Each line indicates the possibility of binding, interaction or complex between two proteins. **C.** The representative Western blot lines of p300 protein in RAW264.7 cells after treatment with BBR in the presence of LPS. β-actin was used as the loading control. **D.** The levels of p300 and CBP expression in peritoneal macrophages of LPS-induced mice after BBR treatment (n = 3).**E.** The representative Western blot lines of p300 protein in peritoneal macrophages of LPS-induced mice after BBR treatment (n = 3). β-actin was used as the loading control. **F.** The representative Western blot lines of p300 protein in white adipose tissue of the high-fat diet-induced obese mice after BBR treatment. β-actin was used as the loading control. **G.** The photomicrographs of p300 expression in white adipose tissue of the high-fat diet-induced obese mice after BBR treatment. **H.** The immunoprecipitation analysis of the binding level of p300 protein to ac-p65^Lys310^ in BMDM cells following BBR treatment in the presence of LPS. The result of the anti-IgG was indicated as a negative control. β-actin was used as the loading control. All data shown are the means ± SEM. *p < 0.05 & **p < 0.01. (For interpretation of the references to color in this figure legend, the reader is referred to the Web version of this article.)

The exact mechanism underlying the BBR-induced reduction in levels of acetylated p65^Lys310^ remains unclear, but previous studies found that acetylases and deacetylases mediate the acetylated levels of p65 [29,30]. Therefore, we examined a variety of acetylases and deacetylases by analyzing the total proteomics as well as the PPI network diagram of p65 in the acetylated proteomics. Among the total proteomics, a list of significantly altered acetylases and deacetylases is identified and presented in Fig. 8A. Of note, p300 is one of the most drastically changed acetylases and deacetylases (Fig. 8A and Supplemental Fig. 5). Moreover, among the predicted upstream acetylases, p300 is known to modulate the acetylation of p65 (Fig. 8B). Consistent with the analysis of the PPI network, an analysis of the available literature highlighted p300 as an interesting candidate acetylase for further study since it increases the levels of acetylated p65^Lys310^ [[31], [32], [33], [34]]. Therefore, we next sought to detect levels of p300 in order to understand the mechanism of BBR modulation of p65^Lys310^ acetylation. *In vitro*, we found that the protein levels of p300 were markedly increased in LPS-stimulated RAW264.7 cells, while the LPS-induced elevation levels of p300 were reversed in BBR-treated cells (Fig. 8C). Similarly, p300 mRNA and protein levels were significantly elevated in peritoneal macrophages of LPS-stimulated acute inflammation mice, but BBR reduced p300 expression in LPS-stimulated acute inflammation mice (Fig. 8D–E). Besides, the mouse model of chronic inflammation with long-term HFD was used in the next experiments. The results showed that the protein levels of p300 in eWAT increased considerably following HFD modeling but decreased after BBR treatment (Fig. 8F–G). These results suggest that BBR could decrease the elevation of p300 expression in macrophages during the occurrence of inflammation.

**Fig. 9:**
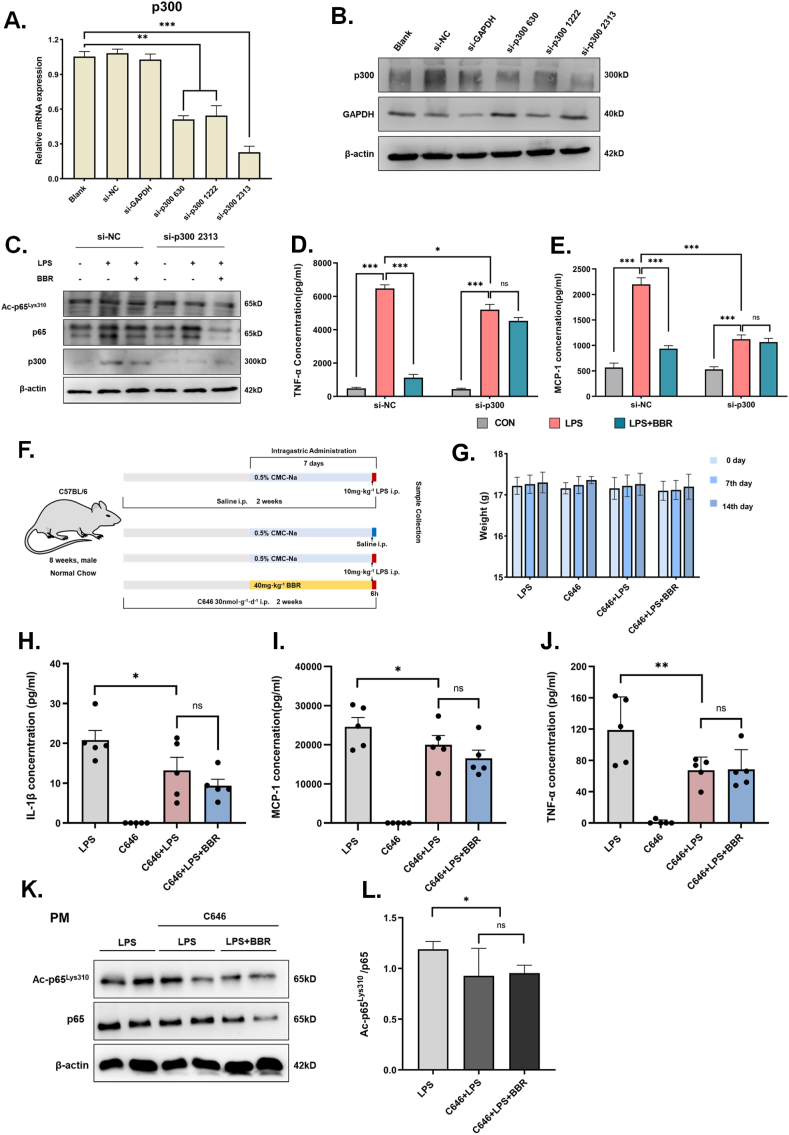
**Effect of BBR on the level of ac-p65Lys310 and inflammation in macrophages with p300 knockdown or inhibitor.** **A-B.** Validation of p300 mRNA and protein levels in RAW264.7 cells after transfection with three different sequences of p300 small interfering RNA (n = 3). The si-NC and si-GAPDH results were used as a negative control. β-actin was used as the loading control. **C.** Representative Western blot lines of ac-p65^Lys310^ and p65 proteins in RAW264.7 cells with p300 knockdown after BBR treatment. β-actin was used as the loading control. The si-NC results were used as a negative control. **D-E.** The levels of TNF-α and MCP-1 secretion in the supernatant of RAW264.7 cells with p300 knockdown after BBR treatment (n = 3). The si-NC results were used as a negative control. **F.** Schematic figure illustrating the animal experiment with C646 treatment. **G.** The changes in body weight after BBR treatment in LPS-induced mice pretreated with C646 (n = 5). **H-J.** The levels of serum IL-1β, MCP-1 and TNF-α after BBR treatment in LPS-induced mice pretreated with C646 (n = 5). **K.** Representative Western blot lines of ac-p65^Lys310^ and p65 proteins after BBR treatment in peritoneal macrophages of LPS-induced mice pretreated with C646. β-actin was used as the loading control. **L.** The protein level of ac-p65^Lys310^ after BBR treatment in peritoneal macrophages of LPS-induced mice pretreated with C646.All data shown are the means ± SEM. *p < 0.05 & **p < 0.01 & ***p < 0.001; ns, no significance.

**Fig. 10:**
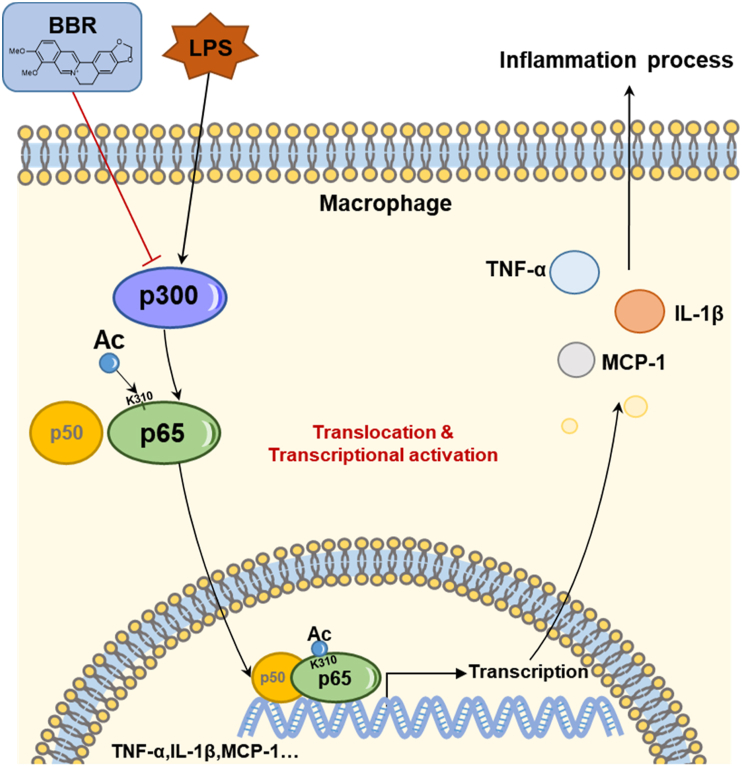
Schematic illustration of the proposed mechanism BBR reduces the level of ac-p65Lys310 to inhibit the translocation and transcription activity of NF-κB through down-regulating the expression of p300, which leads to reversing the inflammatory response in macrophages.

Furthermore, we sought to investigate the role of BBR in the regulation of p300's binding to ac-p65^Lys310^ *in vitro*. The results showed that BBR could weaken the direct binding effect of p300 on ac-p65^Lys310^ in LPS-stimulated BMDM cells (Fig. 8H), suggesting that BBR could inhibit p300 to decrease ac-p65^Lys310^ in LPS-induced macrophages. Moreover, to further examine the inhibitive actions of BBR on ac-p65^Lys310^ activity via downregulation of p300, we knocked down p300 using siRNA and treated cells with BBR in RAW264.7 cells (Fig. 9A–B). As shown in Fig. 9C, the contents of p300 and ac-p65^Lys310^ were markedly reduced in RAW264.7 cells of the siRNA-p300 control group compared with the normal controls, whereas BBR treatment showed a slight change in the contents of p300 and ac-p65^Lys310^ in siRNA-p300 RAW264.7 cells compared with the siRNA-p300 control group, demonstrating that BBR reduces acetylation of p65^Lys310^ through suppressing the expression of p300 (Fig. 9C). Additionally, after the knockdown of p300, fewer inflammatory factors after LPS stimulation were produced by macrophages, and BBR treatment did not produce an obvious effect (Fig. 9D–E). When administering C646, a p300 inhibitor, to a group of acutely inflamed mice, we noticed there was no difference in body weight between the modeling and BBR treatment groups (Fig. 9F–G). In addition, after inhibiting the activity of p300, LPS stimulation resulted in a decrease in the level of serum IL-1β, TNF-α and MCP-1, and the BBR treatment's ability to reduce the level of serum IL-1β, TNF-α and MCP-1 was noticeably diminished (Fig. 9H–J). Similarly, the acetylation of p65 on Lys310 was dramatically reduced in LPS-stimulated macrophages pretreated with a p300 inhibitor, even though the protein level of p65 was not significantly altered (Fig. 9K-L). BBR administration exhibited no appreciable additive effect on the simultaneous acetylation of p65 on Lys310 in LPS-stimulated macrophages pretreated with a p300 inhibitor. Taking these results together, it demonstrates that BBR primarily inhibits p300 to reduce ac-p65^Lys310^ in macrophages (see Fig. 10).

## 4 Discussion

Previous studies have shown that the anti-inflammatory activity of BBR is the most important pharmacological property, which is responsible for its therapeutic roles in multiple inflammatory diseases [5]. In this study, we identified that BBR could weaken the translocation of p65 from the cytoplasm to the nucleus in macrophages through reducing the acetylation of p65 at site Lys310, leading to the decline of NF-κB activity. Furthermore, we elucidated that BBR acts as an epigenetic regulator of p65 at site Lys310 to reduce NF-κB activity by inhibiting p300 in macrophages. Collectively, this study provides direct evidence that BBR could serve as a potential anti-inflammatory drug to treat acute and chronic inflammation-related diseases through inhibiting p300/ac-p65^Lys310^ signaling in macrophages.

Macrophages play a crucial role in tissue hemostasis and pathology during infections and inflammation [18,35]. Macrophages are highly present in inflammatory states and exhibit high levels of pro-inflammatory cytokines like TNF-a, MCP-1 and IL-6 [36]. Concordantly, BBR is believed to play an anti-inflammatory role, and BBR's anti-inflammatory effect is mainly focused on macrophages and is well-described in terms of inflammatory factors secreted by macrophages [6,37]. It has been reported that BBR reduced the elevated levels of serum TNF-α, IL-6 and MCP-1 in obese mice and the expressions of TNF-α, IL-6 and MCP-1 adipose tissues, as well as inhibiting the polarization of M1 in adipose tissue [20,21]. *In vitro*, BBR inhibited the increased expression of TNF-α, IL-6 and MCP-1 in LPS-induced Raw264.7 macrophages [19,21]. Consistent with these findings, we further observed a downregulation of TNF-α, IL-1β and MCP-1 expression in Raw264.7 macrophages and BMDM cells after treatment with BBR under acute and chronic inflammatory conditions, indicating that regulation of macrophage function underlies the mechanism of BBR's therapeutic action.

The p65 subunit of the NF-κB complex in the nucleus of macrophages plays a determinant role in regulating the production of proinflammatory genes and overall inflammatory responses [38]. Of note, the acetylation of p65 determines the strength and duration of the NF-κB-mediated transcriptional response as well as the inflammatory response [39,40]. Chen et al. were the first to propose that acetylated forms of p65 only weakly interacted with IκBα, causing the p65 to translocate to the nucleus quickly [29]. More importantly, earlier research by Chen et al. suggests that acetylation of p65 has a site-specific function in the control of the NF-κB-mediated inflammatory response [29]. Three main acetylation sites have been identified within p65, including lysines 218, 221 and 310 [30]. Of them, acetylation of p65^Lys310^ is necessary for full NF-κB transcriptional activity but has no effect on DNA binding or IB assembly [32,41]. Besides, previous literature has demonstrated that p65 phosphorylation (serines 276 and 536) increases acetylation of the p65^Lys310^, which then increases the transcriptional activity of NF-κB [42]. These findings therefore provide evidence that acetylation of p65^Lys310^ plays an important role in the regulation of NF-κB activity. Previous studies have shown that BBR's anti-inflammatory activity is associated with the regulation of NF-κB activity in macrophages and other types of cells, which includes the decrease of the phosphorylation of IKKβ and NF-κB expression, the inhibition of the translocation of NF-κB into the nucleus, and the reduction of NF-κB DNA binding activity [19,21,[43], [44], [45], [46]]. This study further demonstrates that acetylation of p65 is involved in the suppression of NF-κB activity by BBR through the proteome and acetylated proteome analysis of the acetylation levels of total proteins. Interestingly, acetylation of two lysine sites in p65 with a significant reduction was identified in the LPS-induced macrophages after BBR treatment, which are located at Lys310 and Lys122. BBR could weaken the activity of NF-κB through reducing the acetylation of p65 at site Lys310 to suppress p65 translocation. This trend of lysine acetylation level in p65 seemed similar to our previous result of p65 acetylation level, which was significantly decreased in the LPS-induced macrophages after BBR treatment [19]. Moreover, mutation of p65 at site Lys310 could block BBR-induced reduction of activity of NF-κB, further corroborating that BBR weakens the activity of NF-κB through reducing acetylation of p65 at site Lys310 in macrophages. Altogether, reconstitution of macrophages with wild-type and mutant forms of p65 at site Lys310 revealed that BBR exerts an anti-inflammatory effect through reducing the acetylation of p65 at site Lys310 to suppress the activity of NF-κB.

Whether the acetylation of p65 at Lys310 mediates the anti-inflammatory activity of BBR is further confirmed *in vivo*. In the animal model of acute inflammation, BBR reduced the NF-κB activity and proinflammatory cytokine expression in peritoneal macrophages [46]. Besides, multiple studies have established that BBR treatment reduced activity of NF-κB to inhibit p65 nucleus translocation and expression of proinflammatory cytokines in macrophages from the adipose tissues of obese mouse models [21,47]. As expected, in this study, BBR treatment dramatically decreased the levels of IL-1β, MCP-1, and TNF-α from serum and peritoneal macrophages and eWAT in both LPS-induced acute inflammation mice and HFD-induced chronic inflammation mice, confirming BBR's anti-inflammatory effect in various inflammatory states. Our data corroborated previous studies that BBR reduced serum levels of IL-6 and TNF-α in diet-induced obese mice [21,47]. Furthermore, BBR treatment led to a significant reduction of ac-p65^Lys310^ levels and the p65 NF-κB nucleus translocation in peritoneal macrophages of LPS-stimulated mice and in eWAT of HFD-induced obese mice. These findings elucidate that BBR suppresses nuclear NF-κB function and exerts an anti-inflammatory effect in different inflammatory states via reducing acetylation of p65 at Lys310.

How does BBR reduce the acetylation of p65 at site lys310? We have used a proteome and acetylated proteome screening approach to look for deacetylase and acetylase that mediate BBR-induced down-regulation of ac-p65^Lys310^ in macrophages. Interestingly, among the screened and predicted upstream regulators, only p300 was significantly decreased in BBR-treated macrophages and was attached to BBR and ac-p65^Lys310^ in macrophages, indicating that p300 plays a causal role in BBR-induced down-regulation of ac-p65^Lys310^. p300, a histone acetyltransferase enzyme, mediates downstream signal acetylation of p65 at sites Lys310, Lys218, and Lys221 [31], which are involved in the pro-inflammatory activation of macrophages under high-fat conditions and affect metabolic health in general. Under inflammatory conditions, p300 is significantly upregulated and positively correlated with ac-p65^Lys310^ [32]. A prior investigation revealed that up-regulated p300 expression maintains acetylated p65^Lys310^ levels in the TNF-α-dependent NF-κB signaling pathway in macrophages [33]. Additionally, p300-mediated phosphorylation within the transactivation domain of p65 (S536) is necessary for acetylation of p65^Lys310^ [34]. These studies are consistent with our validated results screened from proteomes and lysine acetylomes. Our present findings showed that BBR inhibited the expression levels of p300 in LPS-stimulated RAW264.7 cells. Concordantly, in this study, we also observed BBR could decrease the elevation of p300 expression in macrophages during the occurrence of inflammation. More importantly, we demonstrate that BBR could weaken the direct binding effect of p300 to ac-p65^Lys310^ in LPS-stimulated BMDM cells from the results of co-immunoprecipitation analysis. Conversely, blocking this association with p300 gene knockdown prevents BBR-induced deacetylation of p65^Lys310^, leading to unchanged p65-dependent inflammatory gene expression. In agreement, when LPS-stimulated acute inflammation mice were preinjected with p300 inhibitors, acetylation of p65^Lys310^ and expression of pro-inflammatory factors in peritoneal macrophages were virtually unchanged after BBR treatment. HFD feeding increased p300 protein levels in the liver of mice, while treatment with the p300-specific inhibitor C646 significantly increased insulin sensitivity and glucose tolerance as well as phosphorylation levels of AKT and GSK3 in the liver of HFD-induced obese mice [48,49]. These results indicate that the inhibition of p300 might be a therapeutic mechanism for the association between berberine and obesity in the adipose tissues of obese individuals. Together, loss of p300 expression occurs in macrophages and blocks BBR-induced deacetylation of p65^Lys310^, which strongly explains a direct mechanistic link between BBR and deacetylation of p65^Lys310^. Therefore, all of these results provide evidence in support of a role for BBR in reducing acetylation of p65^Lys310^ in macrophages by suppressing the expression of p300. However, it is unclear how BBR inhibits p300 expression and will require further investigation in the future.

BBR could decrease the activity of NF-κB and inhibit inflammation via AMPK activation in macrophages [50,51]. BBR also inhibits the activity of NF-κB and inflammatory responses in adipose tissue and RAW264.7 macrophages through increasing the expression of SIRT1 [19,20], which can directly combine with and deacetylate p65 at lysine 310. Additionally, the BBR-induced activation of AMPK phosphorylation in adipocytes and adipose tissue was also attenuated by inhibition or knockout of SIRT1 [20]. These results support the notion that BBR-induced NF-κB inhibition is largely dependent on SIRT1 activation in macrophages. In this study, we further investigate whether SIRT1 and other deacetylases mediate the BBR-induced deacetylation of p65^Lys310^ and the reduction of the transcriptional activity of p65 in macrophages. However, our data show that BBR stimulation does not facilitate the expression of SIRT1 and other deacetylases in LPS-induced RAW264.7 macrophages through the proteome and acetylated proteome analysis of the acetylation level of total proteins, suggesting that SIRT1 and other deacetylases are not involved in BBR-induced deacetylation of p65^Lys310^ of macrophages in this study. Nevertheless, we cannot exclude the possibility that SIRT1 and other deacetylases might be involved in BBR-induced macrophage function *in vitro*. Therefore, the exact role of SIRT1 in BBR-induced macrophage function needs further study.

Our study provides compelling evidence that BBR reduces the acetylation level at Lys310 of p65 to inhibit the translocation and transcription activity of NF-κB through down-regulating the expression of p300, which therefore leads to reversing the inflammatory response in macrophages. Our study elucidates not only a new mechanism for the anti-inflammatory function of BBR in acute-or obesity-induced inflammation but also an effective therapeutic strategy for applications of BBR in inflammation-induced diseases.

## 5 Limitations of the study

In fact, through acetylated proteomics, we caught the changes of two acetylation sites on p65, namely Lys310 and Lys122, which showed the opposite trend. Lys122 is located in the Rel homology domain of p65, adjacent to Lys123. Both acetylation of Lys122 and Lys123 can change the DNA binding ability of NF-κB [31]. The effect of acetylated Lys122/123 is not consistent with the same direction as Lys310, which is similar to that of ‘anti-inflammation’. Our results showed that berberine can promote the acetylation of Lys122. Considering that the role of Lys310 is more critical, we mainly verified and explained the regulation of Lys310 by berberine in this study. Besides, the commercial antibody to Lys122 has not been produced, which also hinders the study of this locus, but that's what we want to explore next. On the other hand, RAW264.7 cells contain endogenous p65 protein, which has some background interference on protein expression and functional verification after lentivirus transfection but does not affect the data analysis and conclusions of this study.

## Author contributions

Shuchen Zhang designed and performed the major experiment in cells and animal experiments and wrote a part of this manuscript. Lingyan Zhou, Pingyuan Xu, Ruonan Zhou, Ziwei Zhu, Yaru Li, Yue Kan and Jiao Li assisted in the performance of cell experiments. Xizhong Yu, Yu Jin and Jing Yan assisted in the performance of animal experiments. Juan Zhao contributed to the performance of immunofluorescence experiment. Wenbin Shang and Penghua Fang contributed in the design of the study, the interpretation of the data and writing of this manuscript. Wenbin Shang supervised the project. All authors read and approved the manuscript.

## Declaration of competing interest

The authors declare no competing interests.

## Data Availability

Data will be made available on request.
