# Supplementary material for: Acetylation of p65Lys310 by p300 in macrophages mediates anti-inflammatory property of berberine
Source: Redox Biol. 2023 Apr 17;62:102704. doi: 10.1016/j.redox.2023.102704 (PMC10172918; doi:10.1016/j.redox.2023.102704)
Supplement: Multimedia component 1 [file mmc1.docx]

**Acetylation of p65Lys310 by p300 in macrophages mediates anti-inflammatory property of berberine**

Supplemental Information

**Supplemental Table 1** Regents and materials.

| **Reagent or resource** | **Identifier** | **Source** |
| --- | --- | --- |
| **Antibodies** | | |
| NF-κB p65 | 8242 | Cell Signaling Technology |
| Acetyl-NF-κB p65 (Lys310) | 12629 | Cell Signaling Technology |
| Acetylated-Lysine | 9441 | Cell Signaling Technology |
| Histone H3 | 4499 | Cell Signaling Technology |
| HDAC 1 | 34589 | Cell Signaling Technology |
| HDAC 2 | 57156 | Cell Signaling Technology |
| HDAC 3 | 85057 | Cell Signaling Technology |
| HDAC 5 | 20458 | Cell Signaling Technology |
| HDAC 6 | 7612 | Cell Signaling Technology |
| SIRT 1 | 9475 | Cell Signaling Technology |
| SIRT 2 | 12650 | Cell Signaling Technology |
| SIRT 7 | 5360 | Cell Signaling Technology |
| Anti-rabbit IgG Antibody | 7074 | Cell Signaling Technology |
| Anti-mouse IgG Antibody | 7076 | Cell Signaling Technology |
| P300 | sc-48343 | Santa Cruz Biotechnology |
| β-actin | sc-47778 | Santa Cruz Biotechnology |
| CD11b Monoclonal Antibody (PE) | 12-0112-82 | Thermo Fisher Scientific |
| F4/80 Monoclonal Antibody (Alexa Fluor 488) | 53-4801-82 | Thermo Fisher Scientific |
| Alexa Fluor 488 goat anti-rabbit antibody | 2145005 | Thermo Fisher Scientific |
| Cy3-labeled Goat Anti-Rabbit IgG | A0516 | Beyotime |
| Protein A-G PLUS-Agarose | sc-2003 | Santa Cruz Biotechnology |
| **Chemicals, peptides, and recombinant proteins** | | |
| Berberine hydrochloride (98% purity) | P1611199 | Adamas-beta |
| Lipopolysaccharide (LPS) (Escherichia coli serotype) | 0111: B4 | Sigma-Aldrich |
| Lipofectamine® RNAiMAX Reagent | 13778 | Invitrogen |
| HiScript ⅡQ RT SuperMix | R222-01 | Vazyme Biotech co.,ltd |
| ChamQ SYBR qPCR Master Mix | Q331-02 | Vazyme Biotech co.,ltd |
| Recombinant mouse M-CSF | abs04383 | Absin |
| Puromycin Dihydrochloride | HY-B1743A | MedChemExpress |
| DAPI | 17510 | Fanbo Biochemicals |
| C646 | HY-13823 | MedChemExpress |
| Carboxymethylcellulose sodium salt, medium viscosity | C9481 | Sigma-Aldrich |
| **Critical commercial assays** |  |  |
| NF-κB p65 Transcription Factor Assay Kit | ab133112 | Abcam |
| Subcellular Protein Fractionation Kit | 78840 | Thermo Fisher Scientific |
| BCA protein assay kit | 23225 | Thermo Fisher Scientific |
| Dulbecco’s modified Eagle’s medium | 11965092 | Thermo Fisher Scientific |
| Fetal bovine serum, qualified, Australia | 10099141 | Thermo Fisher Scientific |
| Cell Counting Kit-8 | CK04 | Dojindo |
| Mouse IL-1β High Sensitivity ELISA Kit | 70-EK201BHS | Multisciences |
| Mouse TNF-a ELISA Kit | 70-EK282/4 | Multisciences |
| Mouse CCL2/MCP-1 ELISA Kit | 70-EK287/2 | Multisciences |

**Supplemental Table 2.** Primer sequences for RT-qPCR

| **Gene** | **GenBank accession** | **Primer sequences** | |
| --- | --- | --- | --- |
| p65 | NC_000085.7 | forward | 5'-ACTGCCGGGATGGCTACTAT-3' |
|  |  | reverse | 5'-TCTGGATTCGCTGGCTAATGG-3' |
| TNF-α | NM_001278601.1 | forward | 5'-ACGGCATGGATCTCAAAGAC-3' |
|  |  | reverse | 5'-GTGGGTGAGGAGCACGTAGT-3' |
| MCP-1 | NM_011333.3 | forward | 5'-CCACAACCACCTCAAGCACT-3' |
|  |  | reverse | 5'-TAAGGCATCACAGTCCGAGTC-3' |
| IL-1β | NC_000068.8 | forward | 5'-GTTCCCATTAGACAACTGC-3' |
|  |  | reverse | 5'-GATTCTTTCCTTTGAGGC-3' |
| CBP | NC_000082.7 | forward | 5'-TTCAGCACTGGTCACAGAGG-3' |
|  |  | reverse | 5'-CAGTCATCACAGCAGCAACC-3' |
| p300 | NC_000081.7 | forward | 5'-CGGTAAAGTGCCTCCAATGT-3' |
|  |  | reverse | 5'-GAAGAACAGCCAAGCACCTC-3' |
| β-Actin | NM_007393.5 | forward | 5'-ACACCCGCCACCACTTCGC-3' |
|  |  | reverse | 5'-TCTGGGCCTCGTCACCCACAT-3' |

**Supplemental Table 3.** The distribution of the number of identified proteins or sites in proteome and acetyl-proteome between groups.

| **Proteomics** | **Compared groups** | **Targets** | **Up-regulated** | **Down-regulated** |
| --- | --- | --- | --- | --- |
| Proteome | LPS / CON | proteins | 445 | 345 |
|  | BBR / CON |  | 34 | 59 |
|  | LPS+BBR / LPS |  | 134 | 368 |
| Acetyl-proteome | LPS / CON | proteins | 218 | 468 |
|  |  | sites | 291 | 688 |
|  | BBR / CON | proteins | 55 | 60 |
|  |  | sites | 87 | 67 |
|  | LPS+BBR / LPS | proteins | 309 | 129 |
|  |  | sites | 420 | 173 |

**Supplemental Table 4.** Protein information enriched in immune processes in acetylation proteome. (A: CON B: LPS C: BBR D: LPS+BBR)

| B vs A：immune system process （ protein：78 , acetylation sites：147） | | | | | | | | | | |
| --- | --- | --- | --- | --- | --- | --- | --- | --- | --- | --- |
| Protein accession | Site | Ratio | Regulated | P value | Protein description | Gene name | A | B | C | D |
| P54987 | 292 | 0.115 | Down | 1.93346E-05 | Cis-aconitate decarboxylase | Acod1 | 2.86 | 0.33 | 3.28 | 0.61 |
| Q7TPR4 | 398 | 0.403 | Down | 9.9274E-07 | Alpha-actinin-1 | Actn1 | 1.50 | 0.60 | 1.31 | 0.67 |
| Q99MU3 | 706 | 0.401 | Down | 0.000041138 | Double-stranded RNA-specific adenosine deaminase | Adar | 1.43 | 0.57 | 1.53 | 0.81 |
| P31230 | 165 | 1.486 | Up | 0.037399 | Aminoacyl tRNA synthase complex-interacting multifunctional protein 1 | Aimp1 | 0.87 | 1.29 | 0.93 | 1.03 |
| Q61490 | 152 | 0.692 | Down | 0.021495 | CD166 antigen | Alcam | 1.25 | 0.87 | 1.34 | 0.85 |
| P10107 | 242 | 0.479 | Down | 0.000038559 | Annexin A1 | Anxa1 | 1.18 | 0.57 | 1.40 | 0.49 |
| Q8R5A3 | 388 | 0.471 | Down | 0.000183077 | Amyloid beta A4 precursor protein-binding family B member 1-interacting protein | Apbb1ip | 1.29 | 0.61 | 1.27 | 1.04 |
| Q9ES28 | 391 | 1.657 | Up | 0.00130228 | Rho guanine nucleotide exchange factor 7 | Arhgef7 | 0.89 | 1.48 | 0.91 | 0.96 |
| Q8BNU0 | 462 | 1.385 | Up | 0.00143631 | Armadillo repeat-containing protein 6 | Armc6 | 0.82 | 1.14 | 1.02 | 1.17 |
| Q62388 | 384 | 1.495 | Up | 0.020604 | Serine-protein kinase ATM | Atm | 0.94 | 1.41 | 0.93 | 0.95 |
| O35143 | 83 | 0.66 | Down | 0.00190273 | ATPase inhibitor, mitochondrial | ATP5IF1 | 1.17 | 0.77 | 0.93 | 1.21 |
| P14211 | 159 | 0.504 | Down | 0.000097464 | Calreticulin | Calr | 1.28 | 0.65 | 1.03 | 0.97 |
|  | 209 | 2.493 | Up | 0.006415 |  |  | 0.79 | 1.96 | 0.69 | 0.86 |
|  | 48 | 0.639 | Down | 0.00027784 |  |  | 1.06 | 0.68 | 0.98 | 1.45 |
|  | 374 | 4.598 | Up | 0.000038579 |  |  | 0.56 | 2.59 | 0.57 | 0.68 |
|  | 153 | 0.458 | Down | 0.000123116 |  |  | 1.26 | 0.58 | 1.08 | 1.01 |
|  | 375 | 3.214 | Up | 0.00181706 |  |  | 0.66 | 2.11 | 0.63 | 1.00 |
|  | 215 | 2.304 | Up | 0.00024046 |  |  | 0.81 | 1.86 | 0.84 | 0.68 |
| P35564 | 228 | 0.64 | Down | 0.0108369 | Calnexin | Canx | 1.21 | 0.77 | 0.95 | 1.16 |
|  | 234 | 0.567 | Down | 0.0044805 |  |  | 1.21 | 0.68 | 1.05 | 1.08 |
|  | 218 | 0.736 | Down | 0.00087519 |  |  | 1.13 | 0.83 | 1.01 | 1.11 |
| P60766 | 153 | 0.296 | Down | 0.000037815 | Cell division control protein 42 homolog | Cdc42 | 1.34 | 0.40 | 1.26 | 0.63 |
| O89053 | 20 | 0.657 | Down | 0.002815 | Coronin-1A | Coro1a | 1.14 | 0.75 | 1.19 | 0.80 |
| Q61093 | 261 | 0.248 | Down | 0.000042656 | Cytochrome b-245 heavy chain | Cybb | 1.33 | 0.33 | 1.59 | 0.31 |
| Q62167 | 81 | 1.493 | Up | 0.0149963 | ATP-dependent RNA helicase DDX3X | Ddx3x | 0.75 | 1.12 | 0.97 | 1.64 |
| Q6Q899 | 859 | 0.497 | Down | 0.0055188 | Probable ATP-dependent RNA helicase DDX58 | Ddx58 | 1.40 | 0.70 | 1.44 | 1.11 |
| Q8R1A4 | 1952 | 1.365 | Up | 0.000195651 | Dedicator of cytokinesis protein 7 | Dock7 | 0.81 | 1.11 | 0.93 | 1.46 |
| P58252 | 598 | 0.674 | Down | 0.00030104 | Elongation factor 2 | Eef2 | 1.32 | 0.89 | 1.05 | 0.52 |
|  | 275 | 1.518 | Up | 1.70902E-05 |  |  | 0.81 | 1.23 | 0.84 | 1.46 |
|  | 498 | 0.594 | Down | 0.004601 |  |  | 1.17 | 0.69 | 1.21 | 0.79 |
|  | 272 | 0.765 | Down | 0.00043655 |  |  | 1.09 | 0.84 | 1.00 | 1.12 |
|  | 572 | 0.638 | Down | 0.0023977 |  |  | 1.23 | 0.78 | 0.97 | 1.01 |
| B2RWS6 | 1553 | 6.293 | Up | 7.9762E-07 | Histone acetyltransferase p300 | Ep300 | 0.34 | 2.15 | 0.88 | 1.00 |
|  | 1768 | 2.493 | Up | 0.000078135 |  |  | 0.64 | 1.59 | 0.93 | 1.15 |
|  | 1673 | 2.158 | Up | 0.000021064 |  |  | 0.64 | 1.39 | 0.77 | 1.71 |
|  | 1703 | 3.585 | Up | 4.6941E-06 |  |  | 0.46 | 1.64 | 0.88 | 1.53 |
|  | 1771 | 2.578 | Up | 3.4886E-06 |  |  | 0.57 | 1.47 | 0.96 | 1.40 |
|  | 1545 | 2.461 | Up | 0.000058553 |  |  | 0.64 | 1.57 | 0.93 | 1.16 |
|  | 1541 | 2.13 | Up | 0.00042047 |  |  | 0.68 | 1.44 | 0.94 | 1.27 |
|  | 1554 | 5.511 | Up | 0.00002123 |  |  | 0.36 | 2.00 | 0.92 | 1.10 |
|  | 1557 | 5.6 | Up | 4.5598E-06 |  |  | 0.36 | 1.99 | 0.87 | 1.23 |
|  | 1589 | 1.403 | Up | 0.00099858 |  |  | 0.80 | 1.12 | 0.98 | 1.43 |
|  | 1548 | 3.183 | Up | 0.000021579 |  |  | 0.55 | 1.76 | 0.91 | 1.09 |
|  | 1550 | 4.125 | Up | 2.4124E-07 |  |  | 0.49 | 2.02 | 0.89 | 0.86 |
|  | 1559 | 4.339 | Up | 0.000037692 |  |  | 0.42 | 1.84 | 0.89 | 1.28 |
|  | 1793 | 1.977 | Up | 0.00029595 |  |  | 0.68 | 1.34 | 0.90 | 1.51 |
|  | 1549 | 3.605 | Up | 4.1213E-06 |  |  | 0.51 | 1.83 | 0.91 | 1.07 |
| Q8CGC7 | 186 | 0.631 | Down | 0.029342 | Bifunctional glutamate/proline--tRNA ligase | Eprs | 1.14 | 0.72 | 1.17 | 0.78 |
| P20491 | 80 | 0.234 | Down | 0.000021837 | High affinity immunoglobulin epsilon receptor subunit gamma | Fcer1g | 2.02 | 0.47 | 2.20 | 0.60 |
| P09528 | 54 | 0.61 | Down | 0.0035436 | Ferritin heavy chain | Fth1 | 1.05 | 0.64 | 1.28 | 0.71 |
| Q91WJ8 | 587 | 0.667 | Down | 0.0038972 | Far upstream element-binding protein 1 | Fubp1 | 1.03 | 0.69 | 1.27 | 0.89 |
| P16858 | 252 | 0.413 | Down | 0.00074376 | Glyceraldehyde-3-phosphate dehydrogenase | Gapdh | 1.21 | 0.50 | 1.29 | 0.68 |
|  | 137 | 0.686 | Down | 0.0053447 |  |  | 1.19 | 0.82 | 0.95 | 0.99 |
| P01902 | 167 | 0.26 | Down | 0.0088834 | H-2 class I histocompatibility antigen, K-D alpha chain | H2-K1 | 1.28 | 0.33 | 1.61 | 0.40 |
| P08103 | 194 | 0.634 | Down | 0.022775 | Tyrosine-protein kinase HCK | Hck | 1.22 | 0.77 | 1.20 | 1.25 |
| P49710 | 60 | 1.336 | Up | 0.0187248 | Hematopoietic lineage cell-specific protein | Hcls1 | 0.96 | 1.28 | 0.96 | 1.01 |
| Q6ZWY9 | 16 | 2.717 | Up | 0.00174273 | Histone H2B type 1-C/E/G | Hist1h2bc | 0.54 | 1.48 | 0.83 | 1.76 |
|  | 6 | 1.902 | Up | 0.0031199 |  |  | 0.66 | 1.26 | 0.88 | 1.78 |
|  | 17 | 2.666 | Up | 0.00078169 |  |  | 0.54 | 1.44 | 0.85 | 1.81 |
|  | 12 | 2.115 | Up | 0.0030618 |  |  | 0.64 | 1.35 | 0.86 | 1.65 |
|  | 21 | 2.277 | Up | 0.00069667 |  |  | 0.59 | 1.35 | 0.88 | 1.77 |
|  | 47 | 0.474 | Down | 0.0127199 |  |  | 1.10 | 0.52 | 1.09 | 1.34 |
|  | 24 | 3.11 | Up | 0.00052404 |  |  | 0.49 | 1.53 | 0.86 | 1.73 |
|  | 13 | 2.739 | Up | 0.00127823 |  |  | 0.54 | 1.48 | 0.83 | 1.77 |
| P30681 | 59 | 1.658 | Up | 0.019763 | High mobility group protein B2 | Hmgb2 | 0.85 | 1.41 | 0.82 | 1.09 |
|  | 30 | 1.77 | Up | 0.00024373 |  |  | 0.87 | 1.54 | 0.78 | 0.93 |
|  | 55 | 2.431 | Up | 0.0008833 |  |  | 0.69 | 1.67 | 0.88 | 0.99 |
|  | 3 | 4.365 | Up | 0.00026109 |  |  | 0.57 | 2.49 | 0.71 | 0.60 |
| P38647 | 138 | 0.637 | Down | 1.9222E-06 | Stress-70 protein, mitochondrial | Hspa9 | 1.21 | 0.77 | 0.95 | 1.17 |
|  | 288 | 0.589 | Down | 0.00042395 |  |  | 1.25 | 0.73 | 0.99 | 1.09 |
|  | 360 | 0.388 | Down | 0.0172641 |  |  | 1.50 | 0.58 | 1.12 | 0.60 |
|  | 567 | 0.677 | Down | 0.00031567 |  |  | 1.13 | 0.77 | 0.90 | 1.40 |
|  | 600 | 0.72 | Down | 0.00027778 |  |  | 1.18 | 0.85 | 1.00 | 1.04 |
|  | 300 | 0.632 | Down | 0.000035624 |  |  | 1.21 | 0.76 | 1.10 | 0.94 |
| P63038 | 233 | 0.331 | Down | 0.00029719 | 60 kDa heat shock protein, mitochondrial | Hspd1 | 1.27 | 0.42 | 1.45 | 0.62 |
|  | 364 | 0.752 | Down | 0.00002446 |  |  | 1.21 | 0.91 | 1.01 | 0.88 |
|  | 91 | 0.749 | Down | 0.00026445 |  |  | 1.19 | 0.89 | 0.88 | 1.17 |
|  | 31 | 0.587 | Down | 1.8518E-06 |  |  | 1.23 | 0.72 | 1.11 | 0.90 |
| Q9BDB7 | 300 | 0.723 | Down | 0.0094418 | Interferon-induced protein 44-like | Ifi44l | 1.14 | 0.83 | 1.20 | 1.34 |
| P24547 | 293 | 0.496 | Down | 0.00204 | Inosine-5'-monophosphate dehydrogenase 2 | Impdh2 | 1.07 | 0.53 | 1.42 | 0.49 |
|  | 109 | 1.758 | Up | 0.035661 |  |  | 0.84 | 1.47 | 0.82 | 1.12 |
| Q9ES52 | 1177 | 2.587 | Up | 0.007539 | Phosphatidylinositol 3,4,5-trisphosphate 5-phosphatase 1 | Inpp5d | 0.67 | 1.73 | 0.73 | 1.13 |
| Q8K4B2 | 66 | 1.635 | Up | 0.029564 | Interleukin-1 receptor-associated kinase 3 | Irak3 | 0.81 | 1.33 | 0.99 | 1.14 |
| P55200 | 2771 | 3.489 | Up | 0.00058089 | Histone-lysine N-methyltransferase 2A | Kmt2a | 0.61 | 2.13 | 0.74 | 0.69 |
| Q61233 | 82 | 0.618 | Down | 0.000176736 | Plastin-2 | Lcp1 | 1.21 | 0.75 | 0.97 | 0.90 |
|  | 472 | 0.687 | Down | 0.00167582 |  |  | 1.08 | 0.74 | 1.09 | 0.95 |
|  | 294 | 0.62 | Down | 0.00046095 |  |  | 1.15 | 0.71 | 1.06 | 0.90 |
| P16045 | 108 | 0.705 | Down | 0.020796 | Galectin-1 | Lgals1 | 1.10 | 0.78 | 1.03 | 0.93 |
| P16110 | 210 | 1.993 | Up | 0.0023167 | Galectin-3 | Lgals3 | 0.84 | 1.68 | 0.89 | 0.69 |
|  | 190 | 0.371 | Down | 0.000142633 |  |  | 1.30 | 0.48 | 1.22 | 0.65 |
| P25911 | 230 | 0.606 | Down | 0.000100726 | Tyrosine-protein kinase Lyn | Lyn | 1.38 | 0.83 | 1.28 | 0.74 |
| P31938 | 104 | 0.749 | Down | 0.0021412 | Dual specificity mitogen-activated protein kinase kinase 1 | Map2k1 | 1.11 | 0.83 | 1.22 | 0.90 |
| P49138 | 75 | 0.732 | Down | 0.000117893 | MAP kinase-activated protein kinase 2 | Mapkapk2 | 1.04 | 0.76 | 1.02 | 1.28 |
| Q08874 | 128 | 2.103 | Up | 0.00113953 | Microphthalmia-associated transcription factor | Mitf | 0.68 | 1.44 | 0.76 | 1.51 |
| P26041 | 79 | 0.76 | Down | 0.0041761 | Moesin | Msn | 1.07 | 0.81 | 1.05 | 1.26 |
|  | 258 | 2.342 | Up | 0.0025833 |  |  | 0.77 | 1.81 | 0.71 | 0.93 |
|  | 253 | 0.737 | Down | 0.00110035 |  |  | 1.13 | 0.83 | 1.09 | 1.07 |
| Q8VDD5 | 29 | 0.435 | Down | 0.0097402 | Myosin-9 | Myh9 | 1.18 | 0.51 | 1.35 | 0.67 |
|  | 860 | 0.682 | Down | 0.0079756 |  |  | 1.15 | 0.78 | 0.98 | 1.08 |
|  | 299 | 0.549 | Down | 0.043158 |  |  | 1.17 | 0.64 | 1.24 | 0.72 |
|  | 228 | 0.649 | Down | 0.0065 |  |  | 1.26 | 0.82 | 0.98 | 0.79 |
|  | 1445 | 1.594 | Up | 0.0084021 |  |  | 0.86 | 1.38 | 0.90 | 1.07 |
| Q8K1X4 | 630 | 0.376 | Down | 0.000119016 | Nck-associated protein 1-like | Nckap1l | 1.39 | 0.52 | 1.26 | 0.67 |
| Q60974 | 1347 | 1.458 | Up | 0.047563 | Nuclear receptor corepressor 1 | Ncor1 | 0.88 | 1.28 | 0.82 | 1.20 |
| Q62433 | 280 | 0.681 | Down | 0.00022388 | Protein NDRG1 | Ndrg1 | 1.02 | 0.69 | 1.04 | 1.01 |
| Q9WTK5 | 741 | 0.732 | Down | 0.0104408 | Nuclear factor NF-kappa-B p100 subunit | Nfkb2 | 1.32 | 0.97 | 0.98 | 1.16 |
| P11928 | 43 | 0.556 | Down | 0.036523 | 2'-5'-oligoadenylate synthase 1A | Oas1a | 1.40 | 0.78 | 1.26 | 1.18 |
| P63005 | 360 | 0.479 | Down | 0.000097135 | Platelet-activating factor acetylhydrolase IB subunit alpha | Pafah1b1 | 1.35 | 0.65 | 1.06 | 0.79 |
| Q8BFV2 | 133 | 1.319 | Up | 0.0122759 | PCI domain-containing protein 2 | Pcid2 | 0.86 | 1.13 | 0.90 | 1.40 |
| P27773 | 218 | 0.736 | Down | 0.0129644 | Protein disulfide-isomerase A3 | Pdia3 | 1.10 | 0.81 | 0.94 | 1.31 |
|  | 94 | 0.27 | Down | 0.0030425 |  |  | 1.45 | 0.39 | 1.32 | 0.48 |
|  | 146 | 2.174 | Up | 0.000536 |  |  | 0.77 | 1.68 | 0.83 | 0.98 |
|  | 366 | 0.522 | Down | 0.00030163 |  |  | 1.26 | 0.66 | 0.99 | 1.11 |
|  | 129 | 0.653 | Down | 0.0009362 |  |  | 1.18 | 0.77 | 1.07 | 0.96 |
|  | 82 | 0.425 | Down | 0.000021534 |  |  | 1.37 | 0.58 | 1.21 | 0.59 |
| Q9CZT4 | 411 | 1.455 | Up | 0.0035014 | DNA-directed RNA polymerase III subunit RPC5 | Polr3e | 0.92 | 1.33 | 0.77 | 1.19 |
| Q0VGB7 | 231 | 1.564 | Up | 0.000076378 | Serine/threonine-protein phosphatase 4 regulatory subunit 2 | Ppp4r2 | 0.79 | 1.24 | 1.02 | 1.06 |
| P35700 | 35 | 0.747 | Down | 0.0056151 | Peroxiredoxin-1 | Prdx1 | 1.18 | 0.88 | 1.12 | 1.00 |
| Q61171 | 119 | 0.38 | Down | 0.00037863 | Peroxiredoxin-2 | Prdx2 | 1.20 | 0.46 | 1.34 | 0.53 |
| Q9QVP9 | 198 | 0.143 | Down | 0.0080017 | Protein-tyrosine kinase 2-beta | Ptk2b | 1.63 | 0.23 | 1.82 | 0.27 |
| P29351 | 522 | 1.317 | Up | 0.0067431 | Tyrosine-protein phosphatase non-receptor type 6 | Ptpn6 | 0.97 | 1.27 | 0.89 | 0.97 |
|  | 277 | 0.728 | Down | 0.0110842 |  |  | 1.13 | 0.82 | 0.92 | 1.22 |
|  | 257 | 0.586 | Down | 0.0049209 |  |  | 1.26 | 0.74 | 0.93 | 1.03 |
| P06800 | 417 | 0.348 | Down | 0.00023699 | Receptor-type tyrosine-protein phosphatase C | Ptprc | 1.23 | 0.43 | 1.65 | 0.46 |
| P61027 | 4 | 1.318 | Up | 0.032684 | Ras-related protein Rab-10 | Rab10 | 0.92 | 1.21 | 0.89 | 1.15 |
| P63001 | 96 | 0.435 | Down | 0.000080888 | Ras-related C3 botulinum toxin substrate 1 | Rac1 | 1.27 | 0.55 | 1.11 | 0.78 |
| P31266 | 201 | 0.456 | Down | 0.020542 | Recombining binding protein suppressor of hairless | Rbpj | 1.26 | 0.57 | 1.31 | 0.64 |
|  | 47 | 2.579 | Up | 0.0070807 |  |  | 0.62 | 1.59 | 0.86 | 1.33 |
| Q04207 | 122 | 0.637 | Down | 0.0096633 | Transcription factor p65 | Rela | 1.15 | 0.73 | 1.03 | 1.15 |
|  | 310 | 1.645 | Up | 0.0034163 |  |  | 0.71 | 1.26 | 1.22 | 0.92 |
| Q8VEE4 | 172 | 1.758 | Up | 0.00168448 | Replication protein A 70 kDa DNA-binding subunit | Rpa1 | 0.79 | 1.39 | 0.94 | 1.12 |
| P63276 | 44 | 0.563 | Down | 0.0078419 | 40S ribosomal protein S17 | Rps17 | 1.22 | 0.69 | 1.19 | 0.73 |
| Q60710 | 378 | 0.731 | Down | 0.00073683 | Deoxynucleoside triphosphate triphosphohydrolase SAMHD1 | Samhd1 | 1.11 | 0.81 | 1.08 | 1.24 |
| Q9CZN7 | 464 | 0.743 | Down | 0.0039828 | Serine hydroxymethyltransferase, mitochondrial | Shmt2 | 1.11 | 0.83 | 0.98 | 1.17 |
|  | 367 | 0.751 | Down | 0.0026392 |  |  | 1.11 | 0.83 | 0.98 | 1.16 |
|  | 302 | 0.715 | Down | 0.00029913 |  |  | 1.30 | 0.93 | 0.91 | 0.83 |
| P08228 | 71 | 1.384 | Up | 0.00025838 | Superoxide dismutase [Cu-Zn] | Sod1 | 0.93 | 1.29 | 0.85 | 0.97 |
| P09671 | 68 | 0.34 | Down | 0.000181439 | Superoxide dismutase [Mn], mitochondrial | Sod2 | 1.64 | 0.56 | 1.57 | 1.03 |
|  | 130 | 0.4 | Down | 0.000098257 |  |  | 1.61 | 0.64 | 1.18 | 1.29 |
| Q8BVK9 | 352 | 0.477 | Down | 0.00075752 | Sp110 nuclear body protein | Sp110 | 1.60 | 0.76 | 1.46 | 0.79 |
|  | 370 | 0.111 | Down | 2.3552E-06 |  |  | 2.16 | 0.24 | 2.30 | 0.40 |
| Q9JM90 | 102 | 0.437 | Down | 0.00049733 | Signal-transducing adaptor protein 1 | Stap1 | 1.50 | 0.66 | 1.46 | 0.66 |
|  | 89 | 0.575 | Down | 0.0155174 |  |  | 1.31 | 0.75 | 1.13 | 1.19 |
| Q62318 | 341 | 0.403 | Down | 4.0862E-06 | Transcription intermediary factor 1-beta | Trim28 | 1.29 | 0.52 | 1.06 | 0.99 |
| Q9JLT4 | 153 | 0.711 | Down | 0.0023169 | "Thioredoxin reductase 2, mitochondrial | Txnrd2 | 1.08 | 0.77 | 0.97 | 1.25 |
| P61089 | 74 | 0.724 | Down | 0.028319 | Ubiquitin-conjugating enzyme E2 N | Ube2n | 1.11 | 0.80 | 1.02 | 1.11 |
| O88342 | 95 | 0.661 | Down | 0.0030961 | WD repeat-containing protein 1 | Wdr1 | 1.19 | 0.79 | 1.02 | 1.06 |
| D vs B：immune system process（protein：48, acetylation sites：91） | | | | | | | | | | |
| Protein accession | Site | Ratio | Regulated Type | P value | Protein description | Gene name | CON | LPS | BBR | LPS+BBR |
| P54987 | 292 | 1.846 | Up | 0.00050066 | Cis-aconitate decarboxylase | Acod1 | 2.86 | 0.33 | 3.28 | 0.61 |
| Q7TPR4 | 436 | 0.547 | Down | 0.0102786 | Alpha-actinin-1 | Actn1 | 1.03 | 1.24 | 1.25 | 0.68 |
| Q99MU3 | 706 | 1.42 | Up | 0.011117 | Double-stranded RNA-specific adenosine deaminase | Adar | 1.43 | 0.57 | 1.53 | 0.81 |
| P31230 | 33 | 1.596 | Up | 0.0065801 | Aminoacyl tRNA synthase complex-interacting multifunctional protein 1 | Aimp1 | 1.03 | 0.92 | 0.86 | 1.46 |
| Q8R5A3 | 388 | 1.712 | Up | 0.007638 | Amyloid beta A4 precursor protein-binding family B member 1-interacting protein | Apbb1ip | 1.29 | 0.61 | 1.27 | 1.04 |
| Q9ES28 | 391 | 0.648 | Down | 0.00183588 | Rho guanine nucleotide exchange factor 7 | Arhgef7 | 0.89 | 1.48 | 0.91 | 0.96 |
| Q62388 | 384 | 0.674 | Down | 0.0046152 | Serine-protein kinase ATM OS=Mus musculus | Atm | 0.94 | 1.41 | 0.93 | 0.95 |
| O35143 | 83 | 1.57 | Up | 0.022796 | ATPase inhibitor, mitochondrial | ATP5IF1 | 1.17 | 0.77 | 0.93 | 1.21 |
| P14211 | 159 | 1.505 | Up | 0.0044986 | Calreticulin | Calr | 1.28 | 0.65 | 1.03 | 0.97 |
|  | 209 | 0.438 | Down | 0.008701 |  |  | 0.79 | 1.96 | 0.69 | 0.86 |
|  | 48 | 2.136 | Up | 0.0002182 |  |  | 1.06 | 0.68 | 0.98 | 1.45 |
|  | 43 | 1.784 | Up | 0.0025821 |  |  | 1.03 | 0.82 | 0.91 | 1.45 |
|  | 374 | 0.262 | Down | 0.00029819 |  |  | 0.56 | 2.59 | 0.57 | 0.68 |
|  | 153 | 1.76 | Up | 0.00053872 |  |  | 1.26 | 0.58 | 1.08 | 1.01 |
|  | 375 | 0.475 | Down | 0.0046174 |  |  | 0.66 | 2.11 | 0.63 | 1.00 |
|  | 215 | 0.366 | Down | 0.000021172 |  |  | 0.81 | 1.86 | 0.84 | 0.68 |
| P35564 | 228 | 1.497 | Up | 0.0113833 | Calnexin | Canx | 1.21 | 0.77 | 0.95 | 1.16 |
|  | 234 | 1.585 | Up | 0.0052636 |  |  | 1.21 | 0.68 | 1.05 | 1.08 |
|  | 171 | 1.368 | Up | 0.028357 |  |  | 0.99 | 1.04 | 0.83 | 1.42 |
|  | 218 | 1.337 | Up | 0.0062382 |  |  | 1.13 | 0.83 | 1.01 | 1.11 |
| P60766 | 153 | 1.581 | Up | 0.0016158 | Cell division control protein 42 homolog | Cdc42 | 1.34 | 0.40 | 1.26 | 0.63 |
| Q62167 | 81 | 1.466 | Up | 0.0133594 | ATP-dependent RNA helicase DDX3X | Ddx3x | 0.75 | 1.12 | 0.97 | 1.64 |
| Q6Q899 | 859 | 1.597 | Up | 0.0134954 | Probable ATP-dependent RNA helicase DDX58 | Ddx58 | 1.40 | 0.70 | 1.44 | 1.11 |
| Q8R1A4 | 1952 | 1.317 | Up | 0.00045719 | Dedicator of cytokinesis protein 7 | Dock7 | 0.81 | 1.11 | 0.93 | 1.46 |
| P58252 | 239 | 1.458 | Up | 0.042182 | Elongation factor 2 | Eef2 | 1.05 | 0.94 | 0.85 | 1.36 |
|  | 598 | 0.584 | Down | 0.039121 |  |  | 1.32 | 0.89 | 1.05 | 0.52 |
|  | 318 | 1.455 | Up | 0.00040165 |  |  | 0.87 | 1.05 | 0.87 | 1.53 |
|  | 272 | 1.344 | Up | 0.00045615 |  |  | 1.09 | 0.84 | 1.00 | 1.12 |
| Q9QZ05 | 1258 | 1.527 | Up | 0.014761 | eIF-2-alpha kinase GCN2 | Eif2ak4 | 0.90 | 0.94 | 0.97 | 1.43 |
| B2RWS6 | 1553 | 0.467 | Down | 0.00027987 | Histone acetyltransferase p300 | Ep300 | 0.34 | 2.15 | 0.88 | 1.00 |
|  | 1768 | 0.723 | Down | 0.0115017 |  |  | 0.64 | 1.59 | 0.93 | 1.15 |
|  | 1545 | 0.74 | Down | 0.00151832 |  |  | 0.64 | 1.57 | 0.93 | 1.16 |
|  | 1554 | 0.55 | Down | 0.00115797 |  |  | 0.36 | 2.00 | 0.92 | 1.10 |
|  | 1557 | 0.617 | Down | 0.00092125 |  |  | 0.36 | 1.99 | 0.87 | 1.23 |
|  | 1548 | 0.617 | Down | 0.00032114 |  |  | 0.55 | 1.76 | 0.91 | 1.09 |
|  | 1550 | 0.424 | Down | 7.6749E-07 |  |  | 0.49 | 2.02 | 0.89 | 0.86 |
|  | 1559 | 0.698 | Down | 0.0192629 |  |  | 0.42 | 1.84 | 0.89 | 1.28 |
|  | 1549 | 0.582 | Down | 0.000024718 |  |  | 0.51 | 1.83 | 0.91 | 1.07 |
| P16858 | 59 | 1.363 | Up | 0.03914 | Glyceraldehyde-3-phosphate dehydrogenase | Gapdh | 1.07 | 0.82 | 1.02 | 1.12 |
|  | 3 | 1.728 | Up | 0.00160098 |  |  | 0.93 | 0.82 | 1.00 | 1.42 |
| P08103 | 194 | 1.626 | Up | 0.0082625 | Tyrosine-protein kinase HCK | Hck | 1.22 | 0.77 | 1.20 | 1.25 |
| Q6ZWY9 | 86 | 2.201 | Up | 0.00083569 | Histone H2B type 1-C/E/G | Hist1h2bc | 0.95 | 0.82 | 0.91 | 1.80 |
|  | 6 | 1.418 | Up | 0.043983 |  |  | 0.66 | 1.26 | 0.88 | 1.78 |
|  | 21 | 1.306 | Up | 0.0071638 |  |  | 0.59 | 1.35 | 0.88 | 1.77 |
|  | 121 | 2.65 | Up | 0.000099285 |  |  | 0.91 | 0.77 | 0.92 | 2.04 |
|  | 47 | 2.58 | Up | 0.0092196 |  |  | 1.10 | 0.52 | 1.09 | 1.34 |
| P30681 | 30 | 0.607 | Down | 0.0082192 | High mobility group protein B2 | Hmgb2 | 0.87 | 1.54 | 0.78 | 0.93 |
|  | 55 | 0.591 | Down | 0.00057758 |  |  | 0.69 | 1.67 | 0.88 | 0.99 |
|  | 3 | 0.239 | Down | 0.0092564 |  |  | 0.57 | 2.49 | 0.71 | 0.60 |
| P38647 | 138 | 1.516 | Up | 0.000119979 | Stress-70 protein, mitochondrial | Hspa9 | 1.21 | 0.77 | 0.95 | 1.17 |
|  | 468 | 1.369 | Up | 0.000039236 |  |  | 1.24 | 0.79 | 0.96 | 1.09 |
|  | 288 | 1.486 | Up | 0.0029786 |  |  | 1.25 | 0.73 | 0.99 | 1.09 |
|  | 567 | 1.82 | Up | 0.000082146 |  |  | 1.13 | 0.77 | 0.90 | 1.40 |
| P63038 | 233 | 1.481 | Up | 0.022577 | 60 kDa heat shock protein, mitochondrial | Hspd1 | 1.27 | 0.42 | 1.45 | 0.62 |
|  | 91 | 1.311 | Up | 0.00101689 |  |  | 1.19 | 0.89 | 0.88 | 1.17 |
| Q9BDB7 | 300 | 1.615 | Up | 0.0073034 | Interferon-induced protein 44-like | Ifi44l | 1.14 | 0.83 | 1.20 | 1.34 |
| Q64345 | 252 | 2.015 | Up | 0.0045009 | Interferon-induced protein with tetratricopeptide repeats 3 | Ifit3 | 0.98 | 0.91 | 0.99 | 1.84 |
| P55200 | 2771 | 0.325 | Down | 0.000181899 | Histone-lysine N-methyltransferase 2A | Kmt2a | 0.61 | 2.13 | 0.74 | 0.69 |
| Q61233 | 328 | 0.706 | Down | 0.031441 | Plastin-2 | Lcp1 | 1.16 | 1.16 | 0.79 | 0.82 |
| P16110 | 210 | 0.411 | Down | 0.00153873 | Galectin-3 | Lgals3 | 0.84 | 1.68 | 0.89 | 0.69 |
|  | 190 | 1.34 | Up | 0.0191968 |  |  | 1.30 | 0.48 | 1.22 | 0.65 |
| P49138 | 75 | 1.683 | Up | 0.0131022 | MAP kinase-activated protein kinase 2 | Mapkapk2 | 1.04 | 0.76 | 1.02 | 1.28 |
| Q08874 | 45 | 0.637 | Down | 0.027578 | Microphthalmia-associated transcription factor | Mitf | 0.34 | 1.73 | 0.55 | 1.10 |
| P26041 | 79 | 1.545 | Up | 0.00186305 | Moesin | Msn | 1.07 | 0.81 | 1.05 | 1.26 |
|  | 258 | 0.51 | Down | 0.00092311 |  |  | 0.77 | 1.81 | 0.71 | 0.93 |
| Q8VDD5 | 860 | 1.384 | Up | 0.0182401 | Myosin-9 | Myh9 | 1.15 | 0.78 | 0.98 | 1.08 |
| P27773 | 218 | 1.616 | Up | 0.0038635 | Protein disulfide-isomerase A3 | Pdia3 | 1.10 | 0.81 | 0.94 | 1.31 |
|  | 146 | 0.582 | Down | 0.002084 |  |  | 0.77 | 1.68 | 0.83 | 0.98 |
|  | 366 | 1.679 | Up | 0.0039644 |  |  | 1.26 | 0.66 | 0.99 | 1.11 |
| Q9CYR6 | 354 | 1.422 | Up | 0.0088386 | Phosphoacetylglucosamine mutase | Pgm3 | 0.96 | 0.85 | 1.04 | 1.21 |
| P29351 | 522 | 0.761 | Down | 0.011776 | Tyrosine-protein phosphatase non-receptor type 6 | Ptpn6 | 0.97 | 1.27 | 0.89 | 0.97 |
|  | 277 | 1.483 | Up | 0.0133361 |  |  | 1.13 | 0.82 | 0.92 | 1.22 |
|  | 257 | 1.389 | Up | 0.027296 |  |  | 1.26 | 0.74 | 0.93 | 1.03 |
| P63001 | 96 | 1.41 | Up | 0.0040556 | Ras-related C3 botulinum toxin substrate 1 | Rac1 | 1.27 | 0.55 | 1.11 | 0.78 |
| Q04207 | 122 | 1.58 | Up | 0.0061179 | Transcription factor p65 | Rela | 1.15 | 0.73 | 1.03 | 1.15 |
| P63276 | 19 | 1.355 | Up | 0.0115781 | 40S ribosomal protein S17 | Rps17 | 1.05 | 0.94 | 0.92 | 1.27 |
| Q60710 | 378 | 1.52 | Up | 0.000059888 | Deoxynucleoside triphosphate triphosphohydrolase SAMHD1 | Samhd1 | 1.11 | 0.81 | 1.08 | 1.24 |
| Q8R0X7 | 431 | 1.439 | Up | 0.048442 | Sphingosine-1-phosphate lyase 1 | Sgpl1 | 1.19 | 0.75 | 1.21 | 1.07 |
|  | 353 | 1.374 | Up | 0.0062443 |  |  | 1.05 | 0.86 | 1.19 | 1.17 |
| Q9CZN7 | 464 | 1.417 | Up | 0.0046189 | Serine hydroxymethyltransferase, mitochondrial | Shmt2 | 1.11 | 0.83 | 0.98 | 1.17 |
|  | 367 | 1.393 | Up | 0.003476 |  |  | 1.11 | 0.83 | 0.98 | 1.16 |
|  | 269 | 1.466 | Up | 0.0115807 |  |  | 1.07 | 0.87 | 0.95 | 1.27 |
| Q60520 | 854 | 0.614 | Down | 0.025703 | Paired amphipathic helix protein Sin3a | Sin3a | 1.01 | 1.18 | 1.05 | 0.73 |
| P08228 | 71 | 0.753 | Down | 0.0020424 | Superoxide dismutase [Cu-Zn] | Sod1 | 0.93 | 1.29 | 0.85 | 0.97 |
| P09671 | 68 | 1.847 | Up | 0.0034025 | Superoxide dismutase [Mn], mitochondrial | Sod2 | 1.64 | 0.56 | 1.57 | 1.03 |
|  | 130 | 1.999 | Up | 0.00022239 |  |  | 1.61 | 0.64 | 1.18 | 1.29 |
| Q9JM90 | 89 | 1.577 | Up | 0.0024406 | Signal-transducing adaptor protein 1 | Stap1 | 1.31 | 0.75 | 1.13 | 1.19 |
| Q62318 | 341 | 1.908 | Up | 0.000003135 | Transcription intermediary factor 1-beta | Trim28 | 1.29 | 0.52 | 1.06 | 0.99 |
| Q9JLT4 | 153 | 1.627 | Up | 0.00043765 | "Thioredoxin reductase 2, mitochondrial | Txnrd2 | 1.08 | 0.77 | 0.97 | 1.25 |
| P61089 | 74 | 1.382 | Up | 0.028661 | Ubiquitin-conjugating enzyme E2 N | Ube2n | 1.11 | 0.80 | 1.02 | 1.11 |
| O88342 | 95 | 1.343 | Up | 0.040698 | WD repeat-containing protein 1 | Wdr1 | 1.19 | 0.79 | 1.02 | 1.06 |

**Supplemental Figure 1.** Induction efficiency of BMDM. Monocytes were isolated from the bone marrow cavity of mouse femur and cultured in DMEM containing 10%FBS with M-CSF for 7 days to induce the differentiation of monocytes into macrophages(**a**). After LPS stimulation(**b**), f4/80 macrophage surface markers and M1 macrophage activation markers CD11b and CD11c were detected by flow cytometry.


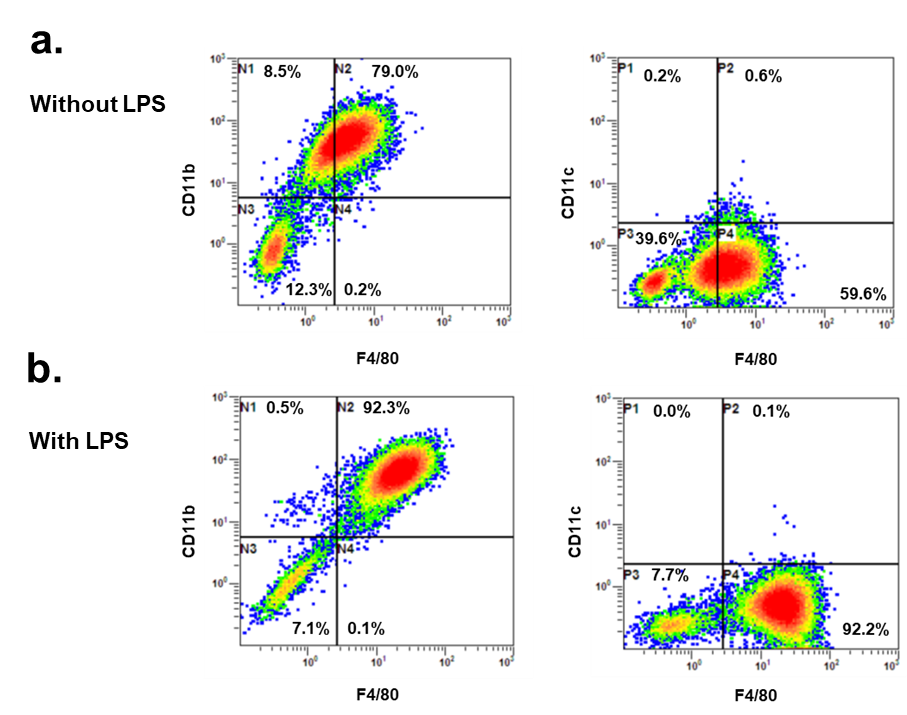


**Supplemental Figure 2.** RAW264.7 cells were transfected with lentivirus. a**.** Schematic diagram of lentivirus transfected cells to construct stable strains. **b.** The transfection efficiency was observed by fluorescence microscope, and the cells successfully transfected expressed EFGP green fluorescent protein.

**
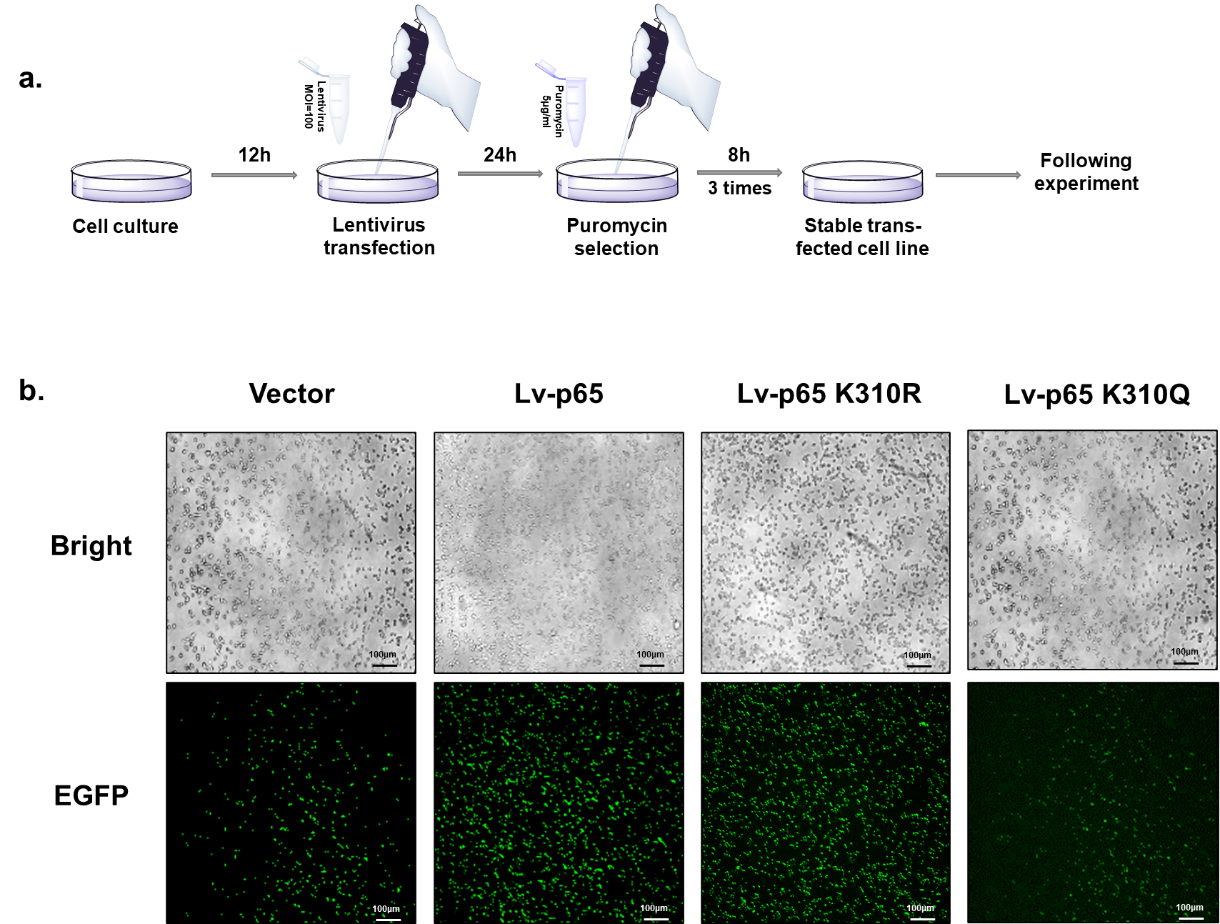
**

**Supplemental Figure 3.** Acetylation modification quantitative omics sample quality control. **a.** The two-dimensional scatter diagram of modified quantitative principal component analysis between repeated samples, in which the better the aggregation degree between repeated samples, the better the quantitative repeatability. **b.** The boxplot of quantitative RSD distribution was modified between repeated samples. The smaller the overall RSD value was, the better the quantitative repeatability was. **c.** Heat map of modified quantitative Pearson correlation coefficient between pairs of samples. This coefficient is a value measuring the degree of linear correlation between two sets of data: the closer Pearson coefficient is to -1, the negative correlation is positive correlation, and the closer Pearson coefficient is to 0, the no correlation. **d.** Subcellular mapping of proteins corresponding to the differential acetylation modification sites. **e.** Protein modification motifs analysis, the highest score of the 3 types of sequences. **f.** COG/KOG functional classification distribution of proteins corresponding to different acetylation modification sites.


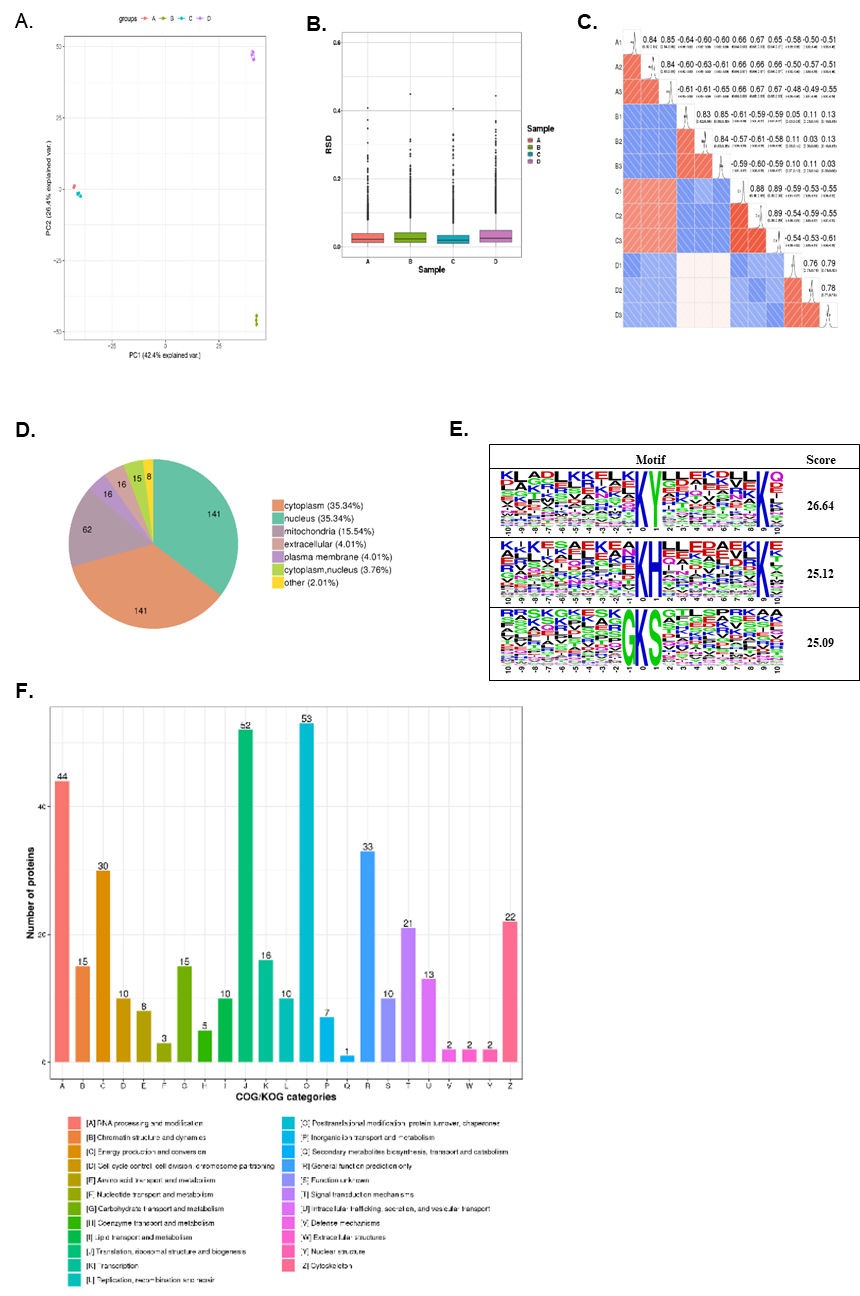


**Supplemental Figure 4.** Each two groups of differential acetyl-proteins were compared and enriched by GO terms annotation via biological process, cellular component and molecular function, and the most enriched pathways were listed below. (**a.** LPS versus CON. **b.** BBR versus CON. **c.** LPS+BBR versus LPS.)


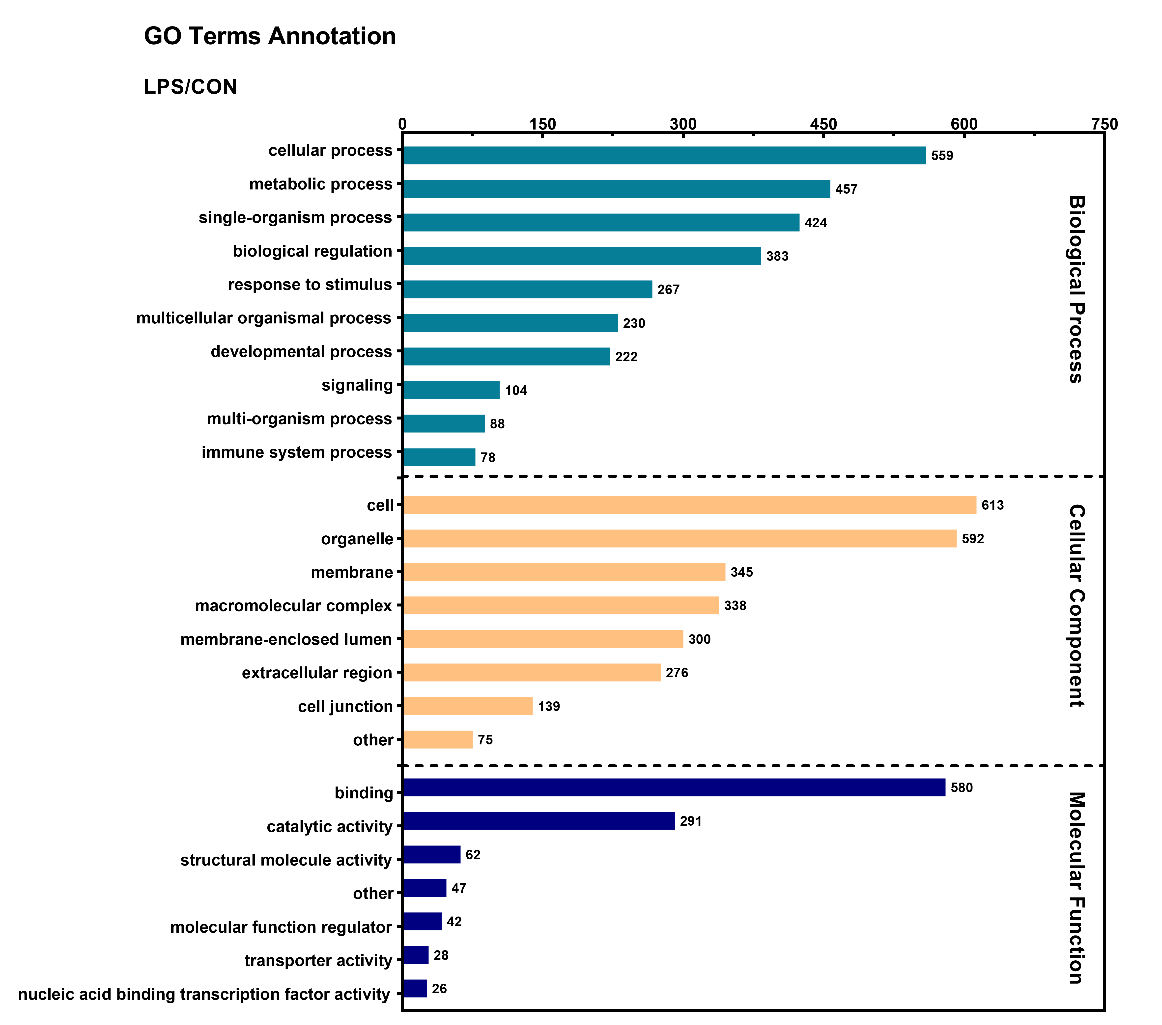

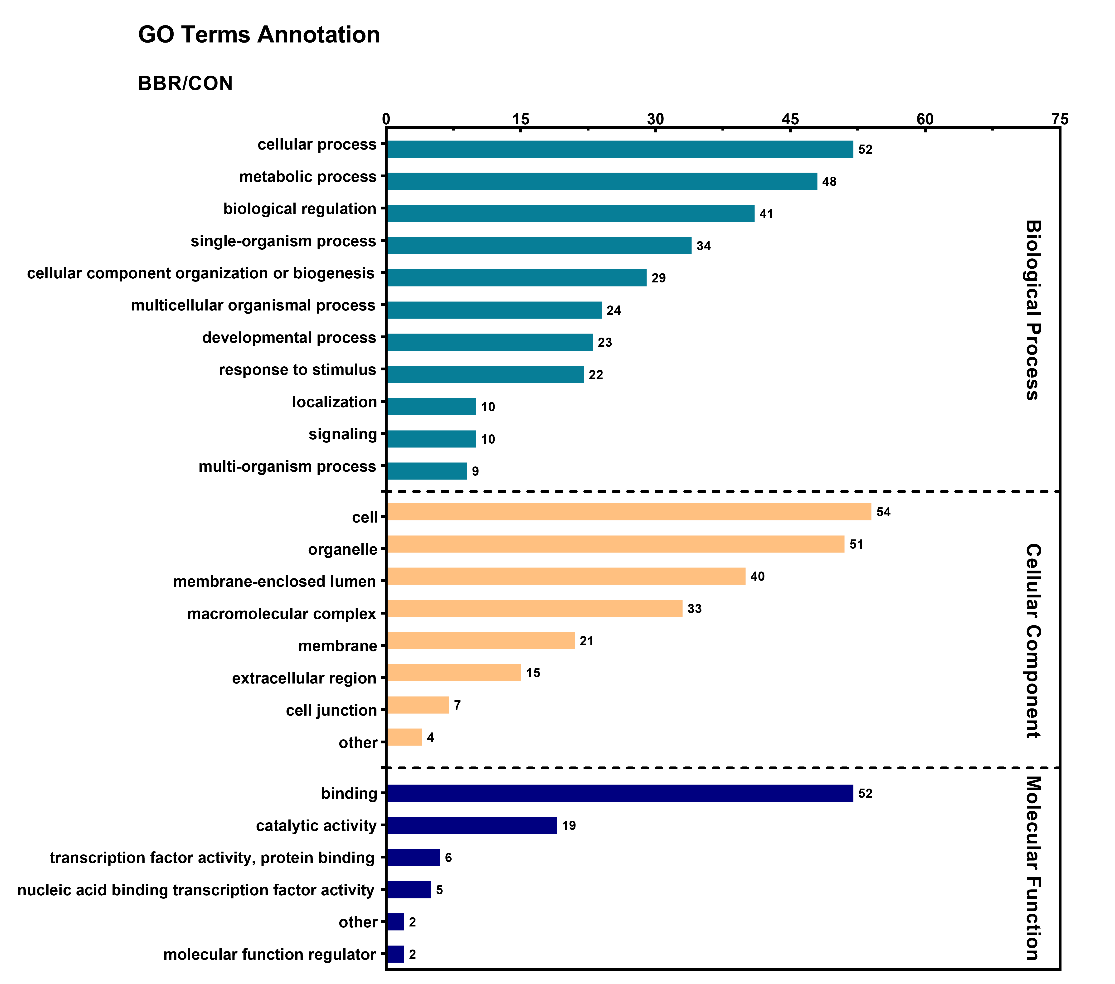

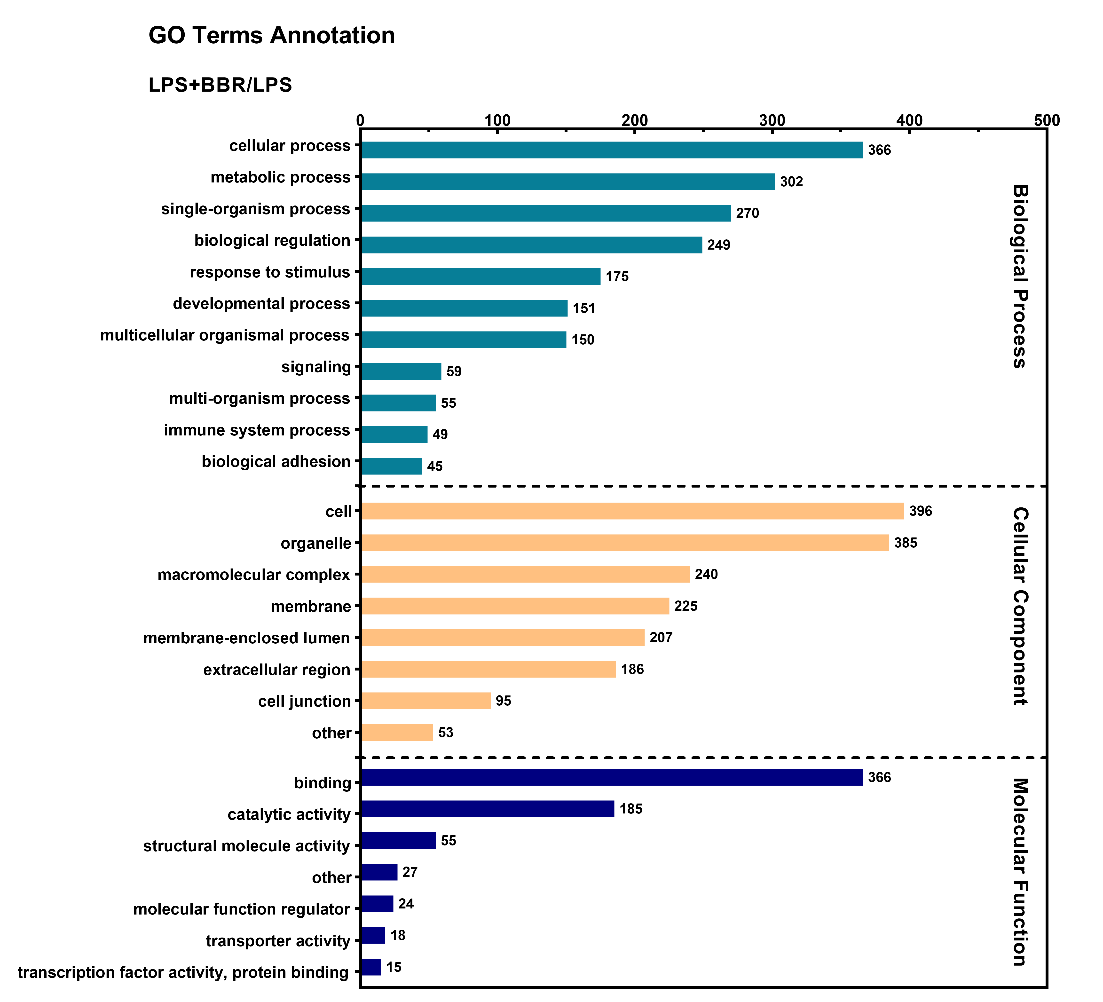


**a.**

**c.**

**b.**

**Supplemental Figure 5.** The protein levels of hdac1-3 were verified by westernblot **(a)** and the gray values **(b)** are shown in the figure.

**
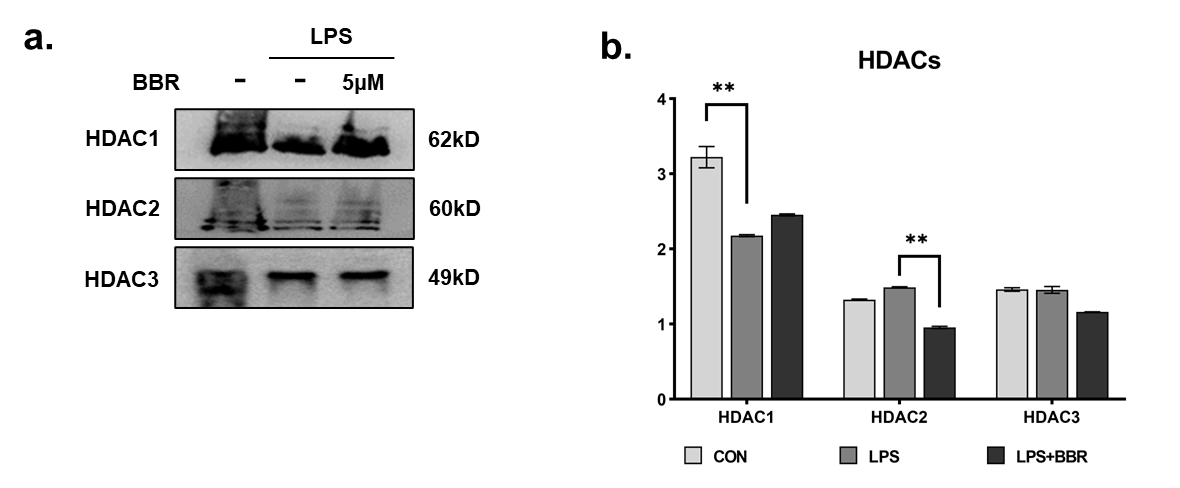
**
